# Epidemiologic Profile of Severe Acute Respiratory Infection in Brazil During the COVID-19 Pandemic: An Epidemiological Study

**DOI:** 10.3389/fmicb.2022.911036

**Published:** 2022-07-01

**Authors:** Nathália Mariana Santos Sansone, Matheus Negri Boschiero, Fernando Augusto Lima Marson

**Affiliations:** ^1^Laboratory of Cell and Molecular Tumor Biology and Bioactive Compounds, São Francisco University, Bragança Paulista, Brazil; ^2^Laboratory of Human and Medical Genetics, São Francisco University, Bragança Paulista, Brazil

**Keywords:** Brazil, COVID-19, COVID-19 underreporting, diagnosis, epidemiology, pandemic, SARS-CoV-2, severe acute respiratory infection

## Abstract

**Background:**

The COVID-19 is a significant public health issue, and monitoring confirmed cases and deaths is an essential epidemiologic tool. We evaluated the features in Brazilian hospitalized patients due to severe acute respiratory infection (SARI) during the COVID-19 pandemic in Brazil. We grouped the patients into the following categories: Influenza virus infection (G1), other respiratory viruses' infection (G2), other known etiologic agents (G3), SARS-CoV-2 infection (patients with COVID-19, G4), and undefined etiological agent (G5).

**Methods:**

We performed an epidemiological study using data from DataSUS (https://opendatasus.saude.gov.br/) from December 2019 to October 2021. The dataset included Brazilian hospitalized patients due to SARI. We considered the clinical evolution of the patients with SARI during the COVID-19 pandemic according to the SARI patient groups as the outcome. We performed the multivariate statistical analysis using logistic regression, and we adopted an Alpha error of 0.05.

**Results:**

A total of 2,740,272 patients were hospitalized due to SARI in Brazil, being the São Paulo state responsible for most of the cases [802,367 (29.3%)]. Most of the patients were male (1,495,416; 54.6%), aged between 25 and 60 years (1,269,398; 46.3%), and were White (1,105,123; 49.8%). A total of 1,577,279 (68.3%) patients recovered from SARI, whereas 701,607 (30.4%) died due to SARI, and 30,551 (1.3%) did not have their deaths related to SARI. A major part of the patients was grouped in G4 (1,817,098; 66.3%) and G5 (896,207; 32.7%). The other groups account for <1% of our sample [G1: 3,474 (0.1%), G2: 16,627 (0.6%), and G3: 6,866 (0.3%)]. The deaths related to SARI were more frequent in G4 (574,887; 34.7%); however, the deaths not related to SARI were more frequent among the patients categorized into the G3 (1,339; 21.3%) and G5 (25,829; 4.1%). In the multivariate analysis, the main predictors to classify the patients in the G5 when compared with G4 or G1-G4 were female sex, younger age, Black race, low educational level, rural place of residence, and the use of antiviral to treat the clinical signs. Furthermore, several features predict the risk of death by SARI, such as older age, race (Black, Indigenous, and multiracial background), low educational level, residence in a flu outbreak region, need for intensive care unit, and need for mechanical ventilatory support.

**Conclusions:**

The possible COVID-19 underreporting (G5) might be associated with an enhanced mortality rate, more evident in distinct social groups. In addition, the patients' features are unequal between the patients' groups and can be used to determine the risk of possible COVID-19 underreporting in our population. Patients with a higher risk of death had a different epidemiological profile when compared with patients who recovered from SARI, like older age, Black, Indigenous, and multiracial background races, low educational level, residence in a flu outbreak region, need for intensive care unit and need for mechanical ventilatory support.

## Introduction

The coronavirus virus family was responsible for several previous epidemics and a significant number of deaths worldwide (Piret and Boivin, 2021; CSR). This virus family accounted for the severe acute respiratory syndrome coronavirus (SARS-CoV) originated in China, whereas the Middle East Respiratory Syndrome Coronavirus (MERS-CoV) originated in Saudi Arabia, and finally, the novel SARS-CoV (SARS-CoV-2) has been responsible for for the Coronavirus Disease (COVID)-19 pandemic. COVID-19 became a major worldwide public health issue, mainly due to the high transmission rate of SARS-CoV-2, which caused the death of millions of people worldwide (Zhu et al., 2020; Piret and Boivin, 2021), including in Brazil, which is considered an epicenter for the disease (Boschiero et al., 2021a,b).

Since the COVID-19 pandemic onset, monitoring the confirmed cases and deaths due to disease is of the utmost importance (Revealing the toll of COVID-19); the association between the highest COVID-19 mortality and with lowest test number is described in the literature, encouraging thorough monitoring of COVID-19 cases (Liang et al., 2020). Other factors, like older age, low educational level, comorbidities, and being a non-White individual, that is, Black, individuals with multiracial background, south Asian, and Indigenous, were also associated with an enhanced mortality rate due to SARS-CoV-2 infection (Hawkins et al., 2020; Santos et al., 2020; Williamson et al., 2020; Yoshikawa and Asaba, 2021; Sansone et al., 2022). Importantly, race plays an essential role in the COVID-19 diagnosis, and some neglected populations might be at even higher risk of underreporting, such as the Indigenous peoples and Black individuals from Brazil, as well as, Black people from the USA (Palamim et al., 2020; Fellows et al., 2021; Mendes et al., 2021). Unfortunately, many countries, including the developed ones, could not test a significant number of individuals and demonstrated an underreporting of COVID-19 cases, such as the USA, Italy, Spain, and especially Brazil (Marson and Ortega, 2020; Carvalho et al., 2021; Kupek, 2021; Lau et al., 2021). According to race, access to SARS-CoV-2 testing is unequal and restrictive for some individuals, especially the Black ones. Some studies showed a lower testing rate in neglected people due to inadequate access to healthcare, fewer testing sites, and long travels to perform a test in regions with minority residents (Lieberman-Cribbin et al., 2020; Rader et al., 2020; Silva et al., 2020; Pletcher et al., 2021). For example, individuals from the Amazon region of Peru, which comprises nearly 265,000 Indigenous individuals (INEI-Perú: Perfil Sociodemográfico), have low access to SARS-CoV-2 testing, increasing the SARS-CoV-2 transmission and mortality due to COVID-19 (Hernández-Vásquez et al., 2021).

Regarding Brazil, several reasons contributed to the underreporting of COVID-19 cases, mainly the limitations to performing widespread SARS-CoV-2 screen by real-time polymerase chain reaction (RT-PCR), operational difficulties in testing all the Brazilian population, and the lack of new tests. Perhaps, these factors contributed to the underreporting, and some severe COVID-19 cases are being diagnosed only as SARI (Bastos et al., 2020; do Prado et al., 2020; Marson, 2020; Carvalho et al., 2021). To registry the Severe Acute Respiratory Infection (SARI) cases after the 2009 H1N1 pandemic, the Brazilian Ministry of Health instituted epidemiological surveillance of respiratory viral agents. The Brazilian government included monitoring SARS-CoV-2 cases in 2020 (Kupek, 2021; SRAG). Unfortunately, Brazil reported more SARI cases without identifying any etiological agent in 2020 and 2021 than compared to previous years (Bastos et al., 2020; SRAG). The non-diagnosis of COVID-19 in individuals with SARI might be troublesome since they would not get proper treatment with specific drugs to treat COVID-19, such as dexamethasone (RECOVERY Collaborative Group et al., 2021). It can also be responsible for disseminating the disease since the prevention measures such as social distancing and wearing masks would not be followed carefully (Advice for the public on COVID-19 – World Health Organization).

In that sense, we evaluated the features (demographic data, hospitalization information, and outcomes) of hospitalized patients with SARI in Brazil, during the COVID-19 pandemic, according to the following groups: SARI due to Influenza virus infection, SARI due to other respiratory viruses' infection, SARI due to other known etiologic agents (OEAs), SARI due to SARS-CoV-2 infection (patients with COVID-19), and SARI due to an undefined etiological agent.

## Materials and Methods

We performed an epidemiological analysis using epidemiologic data available in OpenDataSUS (https://opendatasus.saude.gov.br/) from December 29, 2019, to October 10, 2021. We computed the patients' features using the data from the Brazilian Ministry of Health according to the surveillance data of SARI and from the Information System platform for Epidemiological Surveillance of Influenza (in Portuguese *Sistema de Informação da Vigilância Epidemiológica da Gripe*; SIVEP-Flu). We further divided the patients into two periods. The first period was from December 29, 2019, to December 31, 2020. The second period was from January 1, 2021, to October 10, 2021. A previous study has been published elsewhere (Zeiser et al., 2022) and described two waves in Brazil (February 25, 2020, to April 30, 2021, separated into two waves on November 5, 2020). A wave is characterized by an increasing number of cases until it reaches a peak, followed by a valley period (Salyer et al., 2021). However, we decided two describe our data as two periods (years) to demonstrate the disease evolution according to the temporal development of diagnosis and treatment for COVID-19.

We categorize the patients according to the SARI etiologic: Influenza virus infection, other respiratory viruses' infection, OEAs (known), COVID-19, and undefined etiological agent. In addition, from the dataset, we collected the following patient's features: sex (male and female), age [grouped as follows (years old, y.o.): <1, 1–12, 13–24, 25–60, 61–72, 73–85, and +85], race (White, Black, Asian, individuals with a multiracial background, and Indigenous), educational level (Illiterate, 1^st^ fundamental cycle, 2^nd^ fundamental cycle, High school, and University education), place of residence (Urban, Rural, and Peri-urban), whether the patients live in a flu outbreak region, Flu vaccine status during the last vaccination campaign, treatment for SARI clinical signs with an antiviral drug, need for intensive care unit, need for mechanical ventilatory support (invasive, non-invasive, or not required), closure criterion (laboratory analysis or clinical criteria), and outcome (clinical cure, death due to SARI, or death due to other causes). In our study, we classified the race according to the Brazilian Institute of Geography and Statistics into five official races as described above. The race was self-declared, and the individuals should identify themselves by selecting only one category.

The Brazilian Ministry of Health defines a patient with the severe acute respiratory syndrome (SARS) as “any individual with flu syndrome who also presents: dyspnea/respiratory distress, OR persistent thorax pain, OR O_2_ saturation lower than 95% in ambient air, OR cyanosis” (Saiba como é feita a definição de casos suspeitos de Covid-19 no Brasil). The World Health Organization (WHO) defines it as “a viral respiratory disease caused by a SARS-associated coronavirus” (Severe Acute Respiratory Syndrome (SARS)), such as the patients with COVID-19. This differentiation is essential since the definition of the WHO only comprises those infected by a coronavirus. In contrast, the Brazilian Ministry of Health includes patients infected with any etiological agent causing SARS-like symptoms; in such context, we used the SARI term in our study.

### Statistical Analysis

We performed the statistical analysis using the Statistical Package for the Social Sciences (SPSS) software (IBM SPSS Statistics for Macintosh, Version 27, New York, NY, United States) and OpenEpi software (Dean AG, Sullivan KM, Soe MM. OpenEpi: Open-Source Epidemiologic Statistics for Public Health, Version. www.OpenEpi.com, 2013/04/06). We used the chi-square statistical test to compare the proportion of the individuals with SARI among the different study groups as described before. We calculated the odds ratio (OR) and the 95% confidence interval (95%CI) to estimate the impact of each marker in the different groups according to the SARI classification. We performed the first analysis to compare SARI due to an undefined etiological agent vs. SARS-CoV-2 infection. In addition, we completed the second analysis to associate SARI due to an undefined etiological agent vs. other SARI groups. To facilitate the terminology, in the 2^nd^ analysis, we included the individuals with SARI due to Influenza virus infection, SARI due to other respiratory viruses' infection, SARI due to OEAs (known) and SARI due to SARS-CoV-2 infection (patients with COVID-19) into one category, into one category, namely another SARI group. We also compared the patients' features between both study periods [period 1: December 29, 2019, to December 31, 2020, vs. period 2: January 1, 2021, to October 10, 2021] using the same statistical protocol. We used the OpenEpi software for 2 x 2 tables, including the value for each patient feature, to calculate the OR and the 95%CI. We summarized the results in tables and figures. We used the GraphPad Prism version 8. for Mac (GraphPad Software, San Diego, California USA, www.graphpad.com) to build the figures.

We performed the multivariate analysis using the logistic regression model with the backward stepwise method. We included in the regression model the features with the presence of significant association (*P* ≤ 0.05) in the bivariate model. According to SARI groups, we had the following outcomes in the multivariate analysis: (1^st^ analysis) SARI due to an undefined etiological agent vs. SARS-CoV-2 infection and (2^nd^ analysis) SARI due to an undefined etiological agent vs. other SARI groups. A total of 11 patients' features were included in the multivariate analyses as follows: sex, age, race, educational level, place of residence, whether the patients lived in a flu outbreak region, Flu vaccine status during the last vaccination campaign, treatment for SARI symptoms with an antiviral drug, need for intensive care unit, closure criteria, and outcome. We also performed a third multivariate analysis using all patients' features to determine the main predictors of death. In the logistic regression model, we presented the OR and the 95%CI.

The data used in our study were made publicly available, not containing consent-free personal data since it does not present risks to the research participants.

## Results

### Evolution of the SARI Cases During the COVID-19 Pandemic in Brazil

During the study period comprising the COVID-19 pandemic in Brazil, 2,740,272 patients were hospitalized due to SARI. São Paulo state accounted for the most cases [802,367 (29.3%)] followed by Minas Gerais [298,525 (10.9%)] and Rio de Janeiro [246,823 (9.0%)] states (Table 1). Regarding SARI due to COVID-19, the São Paulo state also accounted for most cases [526,184 (29.0%)], followed by Minas Gerais [177,613 (9.8%)] and Rio de Janeiro [173,093 (9.5%)] states. Whereas, non-COVID-19 SARI, that is, SARI by Influenza, SARI by other respiratory infection, SARI due to OEAs, and SARI due to an undefined etiological agent, was most notified in São Paulo state [267,536 (28.9%)], followed by Minas Gerais [120,912 (13.1%)] and Paraná [74,525 (8.0%)] states (Supplementary Table 1). We described the place of residence of each of the five SARI categories in Supplementary Table 2. Also, in Table 1, we described the number of SARI cases by place of notification per 1,000 inhabitants, and we observed that the higher rate of cases occurred in the Federal District (19.10 cases per 1,000 inhabitants), followed by the São Paulo (17.47 cases per 1,000 inhabitants), Mato Grosso do Sul (17.08 cases per 1,000 inhabitants) states, and Amazonas (12.22 cases per 1,000 inhabitants). For the number of COVID-19 cases by place of notification per 1,000 inhabitants, we had the higher value in the Federal District (14.27 cases per 1,000 inhabitants), followed by the São Paulo (11.46 cases per 1,000 inhabitants), Mato Grosso (12.27 cases per 1,000 inhabitants), and Mato Grosso do Sul (11.38 cases per 1,000 inhabitants) states. Finally, for the number of individuals with SARI due to an undefined etiological agent by place of notification per 1,000 inhabitants, we had the higher value in the Tocantins (6.16 cases per 1,000 inhabitants) state, followed by the Paraná (6.15 cases per 1,000 inhabitants), São Paulo (5.83 cases per 1,000 inhabitants), and Pernambuco (5.78 cases per 1,000 inhabitants) states (Supplementary Table 1). We described the other SARI categories in Supplementary Table 1 for the place of notification; also, we described the cases per 1,000 inhabitants according to the place of residence of each of the five SARI categories in Supplementary Table 2.

**Table 1:** Distribution of the hospitalized patients due to severe acute respiratory infection (SARI) in Brazil during the Coronavirus Disease (COVID)-19 pandemic according to the patients' place of notification and residence.

**State and Federal district**	**Place of** **notification; *N* (%)**	**Place of** **residence; *N* (%)**	**Number of** **inhabitants**	**Individuals by** **place of** **notification per** **1,000 inhabitants**	**Individuals by** **place of residence** **per 1,000** **inhabitants**
Acre	6,640 (0.2%)	6,638 (0.2%)	881,935	7.53	7.53
Alagoas	31,768 (1.2%)	31,978 (1.2%)	3,337,357	9.52	9.58
Amazonas	50,631 (1.8%)	51,723 (1.9%)	4,144,597	12.22	12.48
Amapá	6,318 (0.2%)	6,184 (0.2%)	845,731	7.47	7.31
Bahia	100,737 (3.7%)	101,272 (3.7%)	14,873,064	6.77	6.81
Ceará	111,034 (4.1%)	110,863 (4.0%)	9,132,078	12.16	12.14
Federal district	57,590 (2.1%)	52,506 (1.9%)	3,015,268	19.10	17.41
Espírito Santo	22,389 (0.8%)	22,529 (0.8%)	4,018,650	5.57	5.61
Goiás	90,641 (3.3%)	94,779 (3.5%)	7,018,354	12.91	13.50
Maranhão	33,824 (1.2%)	35,098 (1.3%)	7,075,181	4.78	4.96
Minas Gerais	298,525 (10.9%)	299,457 (10.9%)	21,168,791	14.10	14.15
Mato Grosso do Sul	47,453 (1.7%)	47,872 (1.7%)	2,778,986	17.08	17.23
Mato Grosso	51,465 (1.9%)	52,025 (1.9%)	3,484,466	14.77	14.93
Pará	67,954 (2.5%)	69,026 (2.5%)	8,602,865	7.90	8.02
Paraíba	43,250 (1.6%)	43,264 (1.6%)	4,018,127	10.76	10.77
Pernambuco	105,084 (3.8%)	104,746 (3.8%)	9,557,071	11.00	10.96
Piauí	29,700 (1.1%)	28,609 (1.0%)	3,273,227	9.07	8.74
Paraná	193,060 (7.0%)	192,784 (7.0%)	11,433,957	16.88	16.86
Rio de Janeiro	246,823 (9.0%)	247,101 (9.0%)	17,264,943	14.30	14.31
Rio Grande do Norte	26,380 (1.0%)	26,424 (1.0%)	3,506,853	7.52	7.53
Rondônia	21,021 (0.8%)	21,301 (0.8%)	1,777,225	11.83	11.99
Roraima	4,839 (0.2%)	4,927 (0.2%)	605,761	7.99	8.13
Rio Grande do Sul	148,449 (5.4%)	148,748 (7.0%)	11,377,239	13.05	13.07
Santa Catarina	95,650 (3.5%)	95,540 (3.5%)	7,164,788	13.35	13.33
Sergipe	26,869 (1.0%)	26,555 (1.0%)	2,298,696	11.69	11.55
São Paulo	802,367 (29.3%)	798,382 (29.1%)	45,919,049	17.47	17.39
Tocantins	19,811 (0.7%)	19,598 (0.7%)	1,572,866	12.60	12.46

*We presented the data as the number of individuals (N) and percentage (%)*.

**Figure 1:**
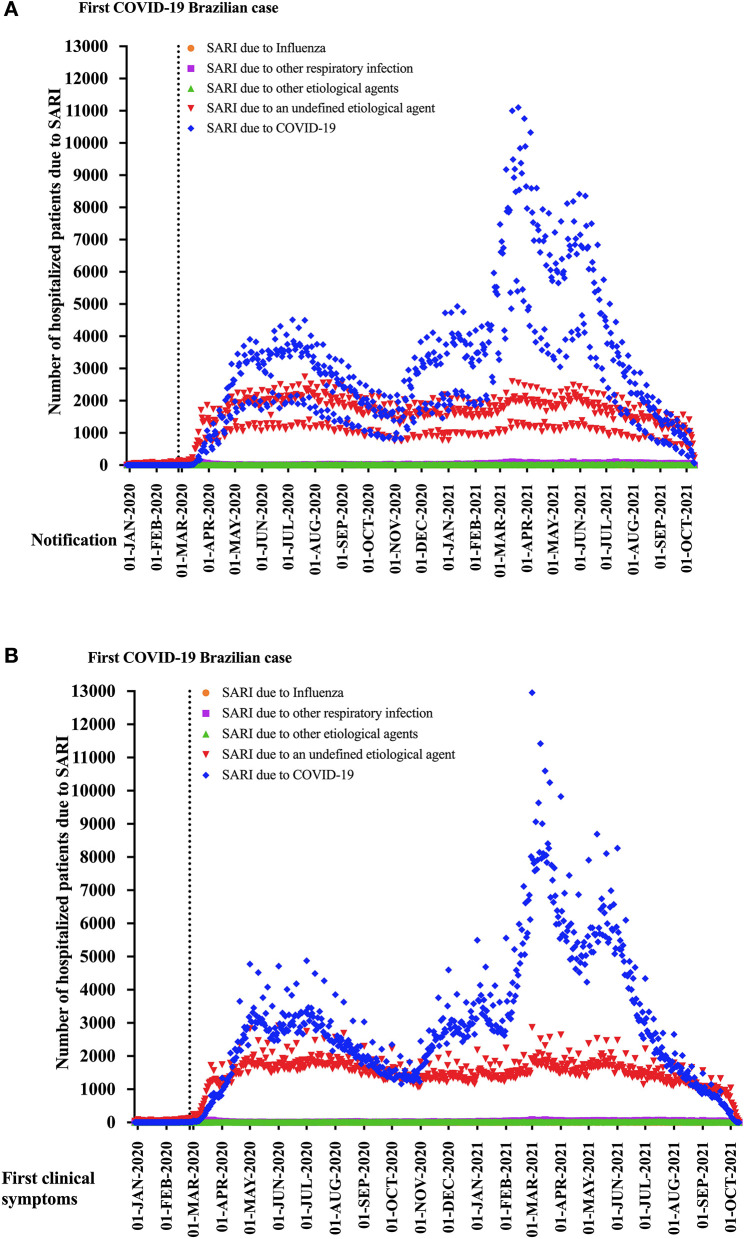
It is shown the distribution of the patients with severe acute respiratory infection (SARI) according to the notification for SIVEP-Flu **(A)** and the onset of clinical symptoms **(B)**. We presented the data according to the SARI categories, which evidenced the increase of the number of COVID-19, as well as the rise of SARI due to an undefined etiological agent among the Brazilian patients after the description of the first positive individual with SARS-CoV-2 infection at São Paulo state.

Figure 1 shows the distribution of the patients with SARI according to the notification for SIVEP-Flu (Figure 1A) and the onset of clinical signs (Figure 1B). We presented the data according to the SARI classification, which evidenced the increase of COVID-19 and the increase of SARI due to an undefined etiological agent among the Brazilian patients after the description of the first positive individual with SARS-CoV-2 infection in São Paulo state in Brazil.

### Patient's Features

In our cohort study, most of the patients with SARI were male (1,495,416; 54.6%) and aged between 25 and 60 years (1,269,398; 46.3%). Also, most of the patients were White (1,105,123; 49.8%), had a high school degree (284,764; 28.7%), lived in an urban area (2,271,009; 94.3%), did not receive the flu vaccine in the last Brazilian campaign (831,051; 74.8%), did not live in a flu outbreak region (655,385; 71.5%), and, most of them also did not use any antiviral drug to treat any clinical signs related to SARI (1,672,295; 88.2%) (Table 2).

**Table 2:** Features of the hospitalized patients due to severe acute respiratory infection (SARI) in Brazil for demographic information, follow-up during the hospitalization, and outcomes during the Coronavirus Disease (COVID)-19 pandemic.

**Patient feature**	**Category**	**N (%)**
Sex	Female	1,245,116 (45.4%)
	Male	1,494,416 (54.5%)
	Missing data	740 (0.1%)
Age	<1 y.o.	61,286 (2.2%)
	1–12 y.o.	114,014 (4.2%)
	13–24 y.o.	75,076 (2.7%)
	25–60 y.o.	1,269,398 (46.3%)
	61–72 y.o.	587,997 (21.5%)
	73–85 y.o.	440,894 (16.1%)
	+85 y.o.	191,607 (7.0%)
Race	White	1,105,123 (40.3%)
	Black	123,504 (4.5%)
	Asian	26,814 (1.0%)
	Individuals from a multiracial background	957,497 (34.9%)
	Indigenous peoples	5,581 (0.2%)
	Missing data	521,753 (19.0%)
Educational level	Illiterate	78,718 (2.9%)
	1^st^ fundamental cycle	258,679 (9.4%)
	2^nd^ fundamental cycle	170,738 (6.2%)
	High school	284,764 (10.4%)
	University education	125,891 (4.6%)
	Not applicable	71,801 (2.6%)
	Missing data	1,749,681 (63.9%)
Place of residence	Urban	2,271,009 (82.9%)
	Rural	126,831 (4.6%)
	Peri-urban	10,052 (0.4%)
	Missing data	332,380 (12.1%)
Living in a Flu outbreak region	Yes	261,469 (9.5%)
	No	655,385 (23.9%)
	Missing data	1,823,418 (66.5%)
Received Flu vaccine	Yes	280,660 (10.2%)
	No	831,051 (30.3%)
	Missing data	1,628,561 (59.5%)
Used antiviral drug to treat the clinical signs	Yes	223,044 (8.1%)
	No	1,672,295 (61.0%)
	Missing data	844,933 (30.9%)
Intensive care unit	Yes	792,754 (28.9%)
	No	1,504,205 (54.9%)
	Missing data	443,313 (16.2%)
Mechanical ventilatory support	Invasive	420,804 (15.4%)
	Non-invasive	1,288,755 (47.0%)
	Not required	562,117 (20.5%)
	Missing data	468,596 (17.1%)
Closure criterion	Laboratorial criterion	2,230,816 (81.4%)
	Clinical - Epidemiological	32,372 (1.2%)
	Clinical	101,384 (3.7%)
	Clinical - Image exams	103,258 (3.8%)
	Missing data	272,442 (9.9%)
Outcome	Cure	1,577,279 (57.6%)
	Death	701,607 (25.6%)
	Death not related to SARI	30,551 (1.1%)
	Missing data	430,835 (15.7%)
SARI categories using the Brazilian Minister of Health definition	SARI due to Influenza	3,474 (0.1%)
	SARI due to other respiratory viruses' infection	16,627 (0.6%)
	SARI due to another known etiological agent	6,866 (0.3%)
	SARI due to an undefined etiological agent	896,207 (32.7%)
	SARI due to COVID-19 (SARS-CoV-2)	1,817,098 (66.3%)

*We presented the data as the number of individuals (N) and percentage (%).*

*SARS-CoV-2, Severe Acute Respiratory Syndrome Coronavirus 2; y.o., years old*.

Most patients with SARI did not need for intensive care unit (1,504,205; 65.5%), whereas 1,288,755 (56.7%) patients needed for non-invasive ventilatory support, 420,804 (18.5%) patients needed for invasive mechanical ventilatory support, and 562,117 (24.7%) patients did not require any mechanical ventilatory support (Table 2). A total of 1,577,279 (68.3%) patients were cured, whereas 701,607 (30.4%) patients died due to the SARI progression, and only 30,551 (1.3%) had their deaths not related to SARI. Most of the hospitalizations were due to COVID-19 (1,817,098; 66.3%), followed by SARI due to an undefined etiological agent (896,207; 32.7%) (Table 2). We showed the patients' features distribution according to the SARI categories in Table 3.

**Table 3:** Association between the severe acute respiratory infection (SARI) categories and the features of the hospitalized patients due to SARI in Brazil during the Coronavirus Disease (COVID)-19 pandemic.

**Patient's features**	**Category**	**SARI due to** **Influenza virus**	**SARI due to other** **respiratory** **viruses' infection**	**SARI due to** **another known** **etiological agent**	**SARI due to an** **undefined** **etiological agent**	**SARI due to** **COVID-19** **(SARS-CoV-2)**	**Total**	***P***
Sex	Female	1,699 (48.9%)	7,619 (45.8%)	3,076 (44.8%)	428,319 (47.8%)	804,403 (44.3%)	1,245,116 (45.4%)	<0.001
	Male	1,772 (51.1%)	9,001 (54.2%)	3,790 (55.2%)	467,446 (52.2%)	1,012,407 (55.7%)	1,494,416 (54.6%)	
Age	<1 y.o.	201 (5.8%)	7,322 (44.0%)	258 (3.8%)	44,783 (5.0%)	8,722 (0.5%)	61,286 (2.2%)	<0.001
	1–12 y.o.	692 (19.9%)	5,918 (35.6%)	534 (7.8%)	91,605 (10.2%)	15,265 (0.8%)	114,014 (4.2%)	
	13–24 y.o.	213 (6.1%)	536 (3.2%)	343 (5.0%)	38,550 (4.3%)	35,434 (2.0%)	75,076 (2.7%)	
	25–60 y.o.	1,404 (40.4%)	1,368 (8.2%)	2,401 (35.0%)	320,694 (35.8%)	943,531 (51.9%)	1,269,398 (46.3%)	
	61–72 y.o.	433 (12.5%)	577 (3.5%)	1,347 (19.6%)	167,494 (18.7%)	418,146 (23.0%)	587,997 (21.5%)	
	73–85 y.o.	358 (10.3%)	599 (3.6%)	1,248 (18.2%)	153,703 (17.2%)	284,986 (15.7%)	440,894 (16.1%)	
	+85 y.o.	173 (5.0%)	307 (1.8%)	735 (10.7%)	79,378 (8.9%)	111,014 (6.1%)	191,607 (7.0%)	
Race	White	1,271 (44.4%)	7,223 (54.6%)	3,319 (53.0%)	335,067 (46.4%)	758,243 (51.4%)	1,105,123 (49.8%)	<0.001
	Black	134 (4.7%)	524 (4.0%)	397 (6.3%)	44,907 (6.2%)	77,542 (5.3%)	123,504 (5.6%)	
	Asian	34 (1.2%)	70 (0.5%)	75 (1.2%)	8,611 (1.2%)	18,024 (1.2%)	26,814 (1.2%)	
	Individuals from a multiracial background	1,422 (49.7%)	5,353 (40.4%)	2,453 (39.2%)	331,031 (45.9%)	617,238 (41.9%)	957,497 (43.2%)	
	Indigenous peoples	3 (0.1%)	70 (0.5%)	21 (0.3%)	1,912 (0.3%)	3,575 (0.2%)	5,581 (0.3%)	
Educational level	Illiterate	147 (8.7%)	1,506 (15.8%)	395 (11.4%)	35,962 (11.2%)	40,708 (6.2%)	78,718 (7.9%)	<0.001
	1^st^ fundamental cycle	344 (20.5%)	607 (6.4%)	1,083 (31.2%)	87,618 (27.3%)	169,027 (25.8%)	258,679 (26.1%)	
	2^nd^ fundamental cycle	190 (11.3%)	343 (3.6%)	822 (23.7%)	48,417 (15.1%)	120,966 (18.5%)	170,738 (17.2%)	
	High school	352 (20.9%)	349 (3.7%)	681 (19.6%)	68,191 (21.3%)	215,191 (32.8%)	284,764 (28.7%)	
	University education	249 (14.8%)	163 (1.7%)	213 (6.1%)	25,431 (7.9%)	99,835 (15.2%)	125,891 (12.7%)	
	Not applicable	399 (23.7%)	6,534 (68.8%)	280 (8.1%)	55,000 (17.2%)	9,588 (1.5%)	71,801 (7.2%)	
Place of residence	Urban	2,973 (93.3%)	13,895 (92.9%)	5,584 (90.8%)	731,535 (93.5%)	1,517,022 (94.7%)	2,271,009 (94.3%)	<0.001
	Rural	202 (6.3%)	547 (3.7%)	517 (8.4%)	46,709 (6.0%)	78,856 (4.9%)	126,831 (5.3%)	
	Peri-urban	12 (0.4%)	514 (3.4%)	47 (0.8%)	3,852 (0.5%)	5,627 (0.4%)	10,052 (0.4%)	
Living in a Flu outbreak region	Yes	292 (17.2%)	880 (19.5%)	488 (18.0%)	91,417 (25.5%)	168,392 (30.7%)	261,469 (28.5%)	<0.001
	No	1,406 (82.8%)	363 (80.5%)	2,219 (82.0%)	267,646 (74.5%)	380,482 (69.3%)	655,385 (71.5%)	
Received Flu vaccine	Yes	487 (29.2%)	1,284 (22.7%)	580 (25.3%)	100,584 (28.5%)	177,725 (23.7%)	280,660 (25.2%)	<0.001
	No	1,182 (70.8%)	4,362 (77.3%)	1,708 (74.7%)	251,894 (71.5%)	571,905 (76.3%)	831,051 (74.8%)	
Used antiviral drug	Yes	1,450 (52.9%)	2,208 (15.1%)	503 (9.6%)	88,925 (14.4%)	129,958 (10.3%)	223,044 (11.8%)	<0.001
to treat the clinical signs	No	1,291 (47.1%)	12,455 (84.9%)	4,730 (90.4%)	527,242 (85.6%)	1,126,577 (89.7%)	1,672,295 (88.2%)	
Intensive care unit	Yes	974 (33.6%)	4,417 (28.2%)	1,848 (31.3%)	199,736 (28.1%)	585,779 (37.5%)	792,754 (34.5%)	<0.001
	No	1,926 (66.4%)	11,249 (71.8%)	4,052 (68.7%)	511,504 (71.9%)	975,474 (62.5%)	1,504,205 (65.5%)	
Mechanical ventilatory support	Invasive	436 (14.9%)	1,542 (9.9%)	1,110 (18.4%)	96,416 (13.7%)	321,300 (20.8%)	420,804 (18.5 %)	<0.001
	Non-Invasive	1,219 (41.6%)	8,878 (57.0%)	3,406 (56.6%)	367,104 (52.1%)	908,148 (58.8%)	1,288,755 (56.7%)	
	Not required	1,273 (43.5%)	5,159 (33.1%)	1,502 (25.0%)	240,438 (34.2%)	313,745 (20.3%)	562,117 (24.7%)	
Closure criterion	Laboratorial criterion	2,990 (90.3%)	16,214 (99.0%)	5,370 (83.6%)	598,654 (88.8%)	1,607,588 (90.9%)	2,230,816 (90.4%)	<0.001
	Clinical - Epidemiological	51 (1.5%)	22 (0.1%)	181 (2.8%)	13,021 (1.9%)	19,097 (1.1%)	32,372 (1.3%)	
	Clinical	214 (6.5%)	136 (0.8%)	509 (7.9%)	57.021 (8.5%)	43.504 (2.5%)	101,384 (4.1%)	
	Clinical - Image exams	55 (1.7%)	4 (0.0%)	366 (5.7%)	5,204 (0.8%)	97,629 (5.5%)	103,258 (4.2%)	
Outcome	Cure	2,662 (85.2%)	14,436 (94.8%)	4,272 (68.1%)	478,039 (76.1%)	1,077,870 (65.1%)	1,577,279 (68.3%)	<0.001
	Death	444 (14.2%)	696 (4.6%)	1,339 (21.3%)	124,241 (19.8%)	574,887 (34.7%)	701,607 (30.4%)	
	Death not related to SARI	19 (0.6%)	102 (0.7%)	664 (10.6%)	25,829 (4.1%)	3,937 (0.2%)	30,551 (1.3%)	

*We presented the data as the number of individuals (N) and percentage (%).*

*We did the statistical analyses using the chi-square test. We adopted an alpha error of 0.05.*

*SARS-CoV-2, Severe Acute Respiratory Syndrome Coronavirus 2; y.o., years old*.

### Bivariate Analysis of the Patients' Features Associated With SARI Due to an Undefined Etiological Agent vs. SARI Due to COVID-19

In our study, female individuals were more likely to be diagnosed with SARI due to an undefined etiological agent than COVID-19 (OR: 1.153; 95%CI: 1.147–1.159) (Supplementary Table 3). In the same way, younger individuals were also more likely to be diagnosed with SARI due to an undefined etiological agent than COVID-19, which is aged <1 y.o. (OR: 15.1; 95%CI: 14.76–15.46), between 1 and 12 y.o. (OR: 17.6; 95%CI: 17.35–17.97) and 13–24 y.o. (OR: 3.201; 95%CI: 3.153–3.249), were at increased risk when compared to individuals between 25 and 60 y.o. (Supplementary Table 3). Even though older individuals, those aged between 61 and 72 y.o. (OR: 1.179; 95%CI: 1.17–1.187), 73–85 y.o. (OR: 1.587; 95%CI: 1.575–1.599), and +85 y.o. (OR: 2.104; 95%CI: 2.083–2.125) (Supplementary Table 3) also presented an enhanced chance of being diagnosed with SARI due to an undefined etiological agent when compared to COVID-19.

Most of the individuals from the neglected races such as Black (OR: 1.311; 95%CI: 1.285–1.327), Asian (OR: 1.081; 95%CI: 1.053–1.11), individuals with a multiracial background (OR: 1.214; 95%CI: 1.207–1.221), and Indigenous peoples (OR: 1.21; 95%CI: 1.145–1.28), were more likely to be diagnosed with SARI due to an undefined etiological agent than COVID-19 when compared to White individuals (Supplementary Table 3). In the same way, individuals with decreased educational levels also presented a higher chance of being diagnosed with SARI due to an undefined etiological agent. For instance, the illiterate presented a 2.5-fold increase (OR: 3.468; 95%CI: 3.4–3.537), and those with only the 1^st^ fundamental cycle presented a 1-fold-increase (OR: 2.035; 95%CI: 2.003–2.068), those with the 2^nd^ fundamental cycle presented a 0.6-fold-increase (OR: 1.571; 95%CI: 1.544–1.599), and those with a high school diploma presented a 0.2-fold-increase (OR: 1.244; 95%CI: 1.224–1.264) when compared to those who finished a college (Supplementary Table 3).

Individuals with SARI due to an undefined etiological agent were less likely to need invasive (OR: 0.392; 95%CI: 0.388–0.395) and non-invasive (OR: 0.528; 95%CI: 0.524–0.531) mechanical ventilatory support when compared to patients with COVID-19. Individuals with SARI due to an undefined etiological agent were also less likely to need an intensive care unit (OR: 0.65; 95%CI: 0.646–0.654) and die (OR: 0.487; 95%CI: 0.484–0.491) when compared to the patients with COVID-19. However, they presented a higher chance of death not related to SARI (OR: 14.79; 95%CI: 14.3–15.3) when compared to patients with COVID-19 (Supplementary Table 3).

**Figure 2:**
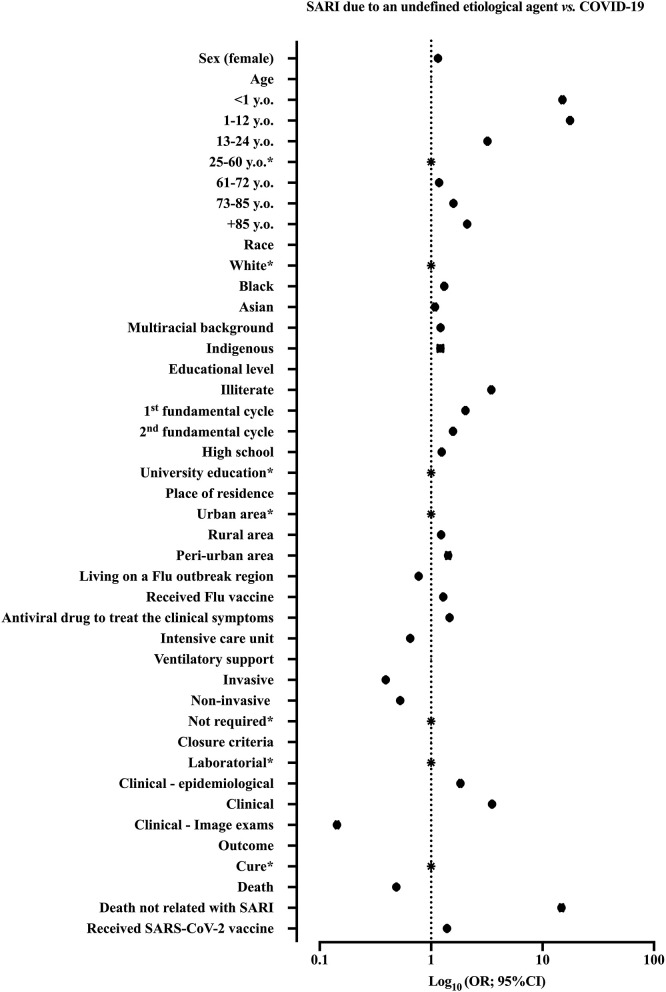
We demonstrated the result of bivariate analysis to identify the patients' features associated with severe acute respiratory infection (SARI) diagnosis. We compared the patients with SARI due to an undefined etiological agent vs. COVID-19. We demonstrated the odds ratios (ORs) and 95% confidence interval (95%CI). SARS-CoV-2, Severe Acute Respiratory Syndrome Coronavirus 2; y.o., years old. *Reference group.

We summarized the patients' features from the bivariate analysis in Supplementary Table 3. We showed the ORs and 95%CI in Figure 2.

### Bivariate Analysis of the Patients' Features Associated With SARI Due to an Undefined Etiological Agent vs. Other SARI Patients

Female individuals were more likely to be diagnosed with SARI due to an undefined etiological agent than other SARI patients (OR: 1.152; 95%CI: 1.146–1.158) (Supplementary Table 3). In the same way, younger individuals [aged <1 y.o. (OR: 8.028; 95%CI: 7.882–8.176), between 1 and 12 y.o. (OR: 12.09; 95%CI: 11.91–12.28), and 13–24 y.o. (OR: 3.122; 95%CI: 3.076–3.169)] when compared to individuals aged between 25 and 60 y.o. were more likely to be diagnosed with SARI due to an undefined etiological agent than other SARI patients (Supplementary Table 3). Even though older individuals, those aged between 61 and 72 y.o. (OR: 1.178; 95%CI: 1.17–1.187), 73–85 y.o. (OR: 1.583; 95%CI: 1.572–1.595), and +85 y.o. (OR: 2.092; 95%CI: 2.072–2.113) (Supplementary Table 3) also presented an enhanced chance of being diagnosed with SARI due to an undefined etiological agent when compared to other SARI patients aged between 25 and 60 y.o.

Individuals with SARI due to an undefined etiological agent were less likely to need both invasive (OR: 0.398; 95%CI: 0.394–0.401) and non-invasive (OR: 0.533; 95%CI: 0.529–0.536) mechanical ventilatory support when compared to other SARI patients. Individuals with SARI due to an undefined etiological agent were less likely to need an intensive care unit (OR: 0.654; 95%CI: 0.65–0.658) and die due to SARI progression (OR: 0.495; 95%CI: 0.491–0.498) when compared to patients with COVID-19. However, they also presented a higher chance of death not related to SARI (OR: 12.58; 95%CI: 12.19–12.98) (Supplementary Table 3) when compared with patients with COVID-19.

Most of the individuals from the neglected races such as Black (OR: 1.313; 95%CI: 1.297–1.329), individuals with a multiracial background (OR: 1.214; 95%CI: 1.207–1.222), and Indigenous peoples (OR: 1.198; 95%CI: 1.133–1.266), were more likely to be diagnosed with SARI due to an undefined etiological agent than other SARI when compared to White individuals (Supplementary Table 3). In the same way, individuals with decreased educational levels also presented a higher chance of being diagnosed with SARI due to an undefined etiological agent. For instance, the illiterate presented a 2.3-fold increase (OR: 3.323; 95%CI: 3.258–3.389), and those with only the 1^st^ fundamental cycle presented a 1-fold-increase (OR: 2.023; 95%CI: 1.991–2.056), those with the 2^nd^ fundamental cycle presented a 0.6-fold-increase (OR: 1.564; 95%CI: 1.537–1.591), and those with a high school diploma presented a 0.2-fold-increase (OR: 1.244; 95%CI: 1.224–1.264) when compared to those who finished a college (Supplementary Table 3).

**Figure 3:**
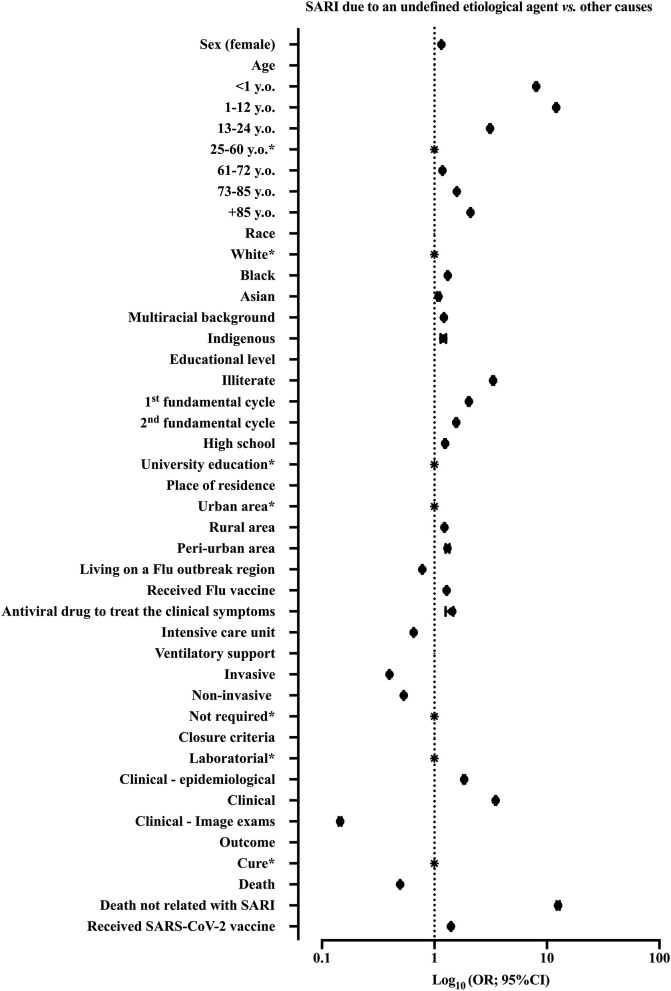
We demonstrated the result of bivariate analysis to identify the patients' features associated with severe acute respiratory infection (SARI) diagnosis. We compared the patients with SARI due to an undefined etiological agent vs. other causes. The other causes included SARI due to Influenza virus infection, SARI due to other respiratory viruses' infection, SARI due to other known etiological agents, and SARI due to SARS-CoV-2 infection (patients with COVID-19). We demonstrated the odds ratios (ORs) and 95% confidence interval (95%CI). SARS-CoV-2, Severe Acute Respiratory Syndrome Coronavirus 2; y.o., years old. *Reference group.

We summarized the patients' features from the bivariate analysis in Supplementary Table 3. We showed the ORs and 95%CI presented in Figure 3.

### Multivariate Analysis

We performed the multivariate analysis using binary logistic regression to determine whether the patients' features could categorize the individuals according to SARI groups. We compared the patients for (primary analysis) SARS-CoV-2 infection vs. SARI due to an undefined etiological agent and (secondary analysis) SARI due to an undefined etiological agent vs. another SARI group. We built two models for each analysis, including the following patient features: (model 1) sex, age, race, educational level, place of residence, whether the patients live in a flu outbreak region, Flu vaccine status during the last vaccination campaign, treatment for SARI symptoms with an antiviral drug, need for intensive care unit, closure criteria, and outcome; (model 2) sex, age, race, educational level, place of residence, whether the patients live in a flu outbreak region, and Flu vaccine status during the last vaccination campaign. We showed the complete information in Table 4, Supplementary Table 4, and Supplementary Table 5. In addition, we did a third analysis to describe the main predictors of death in patients with SARI (Table 5).

**Table 4:** Multivariate analysis using Binary Logistic Regression for the association between the severe acute respiratory infection (SARI) categories and the features of the hospitalized patients due to SARI in Brazil during the Coronavirus Disease (COVID)-19 pandemic.

**Patients' features**	**Patients with SARI due to an undefined etiological** **agent vs. patients with COVID-19**				**Patients with SARI due to an undefined etiological** **agent vs. patients with other causes of SARI**			
	***P***	**OR**	**95% CI for OR**		***P***	**OR**	**95% CI for OR**	
			**Lower**	**Upper**			**Lower**	**Upper**
**Model 1**. Included social and demographic data as well as hospitalization information ^a^								
Sex (female)	<0.001	1.116	1.093	1.139	<0.001	1.112	1.089	1.135
Age								
<1 y.o.	<0.001	7.556	6.557	8.709	<0.001	5.123	4.557	5.759
1–12 y.o.	<0.001	9.156	8.448	9.923	<0.001	6.563	6.113	7.046
13–24 y.o.	<0.001	3.571	3.379	3.774	<0.001	3.401	3.221	3.591
25–60 y.o.	<0.001				<0.001			
61–72 y.o.	0.088	1.025	0.996	1.055	0.070	1.027	0.998	1.056
73–85 y.o.	<0.001	1.438	1.392	1.485	<0.001	1.435	1.390	1.481
+85 y.o.	<0.001	2.091	2.002	2.183	<0.001	2.070	1.983	2.161
Race								
White	<0.001				<0.001			
Black	<0.001	1.224	1.172	1.279	<0.001	1.223	1.172	1.277
Asian	0.888	1.008	0.908	1.118	0.861	1.009	0.910	1.119
Individuals from a multiracial background	<0.001	0.939	0.918	0.961	<0.001	0.938	0.917	0.960
Indigenous peoples	<0.001	0.333	0.276	0.402	<0.001	0.372	0.310	0.446
Educational level								
Illiterate	<0.001	2.186	2.077	2.301	<0.001	2.161	2.054	2.273
1^st^ fundamental cycle	<0.001	1.858	1.788	1.930	<0.001	1.855	1.786	1.927
2^nd^ fundamental cycle	<0.001	1.605	1.543	1.671	<0.001	1.606	1.544	1.671
High school	<0.001				<0.001			
High school	<0.001	1.246	1.201	1.294	<0.001	1.252	1.206	1.299
Not applicable	<0.001	2.151	1.915	2.417	<0.001	1.660	1.504	1.833
Place of residence								
Urban	<0.001				<0.001			
Rural	<0.001	1.257	1.202	1.314	<0.001	1.261	1.207	1.318
Peri-urban	0.200	1.126	0.939	1.349	0.106	1.155	0.970	1.374
Living in a Flu outbreak region	<0.001	0.706	0.688	0.724	<0.001	0.720	0.702	0.738
Received Flu vaccine	<0.001	0.931	0.911	0.952	<0.001	0.937	0.917	0.958
Used antiviral drug to treat the clinical signs	<0.001	1.376	1.341	1.412	<0.001	1.312	1.280	1.346
Need for intensive care unit	<0.001	0.879	0.857	0.902	<0.001	0.875	0.853	0.898
Closure criterion								
Laboratorial criterion	<0.001				<0.001			
Clinical – Epidemiological	<0.001	1.277	1.136	1.434	<0.001	1.307	1.165	1.467
Clinical	<0.001	4.307	4.002	4.635	<0.001	4.252	3.956	4.570
Clinical – Image exams	<0.001	0.108	0.094	0.125	<0.001	0.112	0.097	0.128
Outcome								
Clinical cure	<0.001				<0.001			
Death	<0.001	0.445	0.433	0.458	<0.001	0.452	0.439	0.465
Death not related to SARI	<0.001	14.350	12.595	16.349	<0.001	12.546	11.099	14.182
Constant	<0.001	0.339			<0.001	0.336		
**Model 2**. Included social and demographic data ^b^								
Sex (female)	<0.001	1.130	1.110	1.150	<0.001	1.126	1.107	1.146
Age								
<1 y.o.	<0.001	6.603	5.833	7.474	<0.001	4.766	4.295	5.289
1–12 y.o.	<0.001	7.782	7.265	8.334	<0.001	6.007	5.646	6.390
13–24 y.o.	<0.001	2.861	2.736	2.993	<0.001	2.773	2.652	2.899
25–60 y.o.	<0.001				<0.001			
61–72 y.o.	<0.001	0.893	0.872	0.914	<0.001	0.896	0.875	0.918
73–85 y.o.	<0.001	1.104	1.076	1.134	<0.001	1.109	1.080	1.139
+85 y.o.	<0.001	1.438	1.387	1.490	<0.001	1.438	1.388	1.491
Race								
White	<0.001				<0.001			
Black	<0.001	1.218	1.175	1.264	<0.001	1.217	1.174	1.262
Asian	0.622	0.979	0.900	1.065	0.640	0.980	0.901	1.066
Individuals from a multiracial background	<0.001	0.955	0.937	0.973	<0.001	0.957	0.940	0.975
Indigenous peoples	<0.001	0.368	0.317	0.429	<0.001	0.403	0.348	0.467
Educational level								
Illiterate	<0.001	2.018	1.935	2.104	<0.001	1.997	1.915	2.082
1^st^ fundamental cycle	<0.001	1.707	1.654	1.761	<0.001	1.702	1.649	1.756
2^nd^ fundamental cycle	<0.001	1.426	1.380	1.474	<0.001	1.425	1.379	1.473
High school	<0.001	1.167	1.132	1.204	<0.001	1.170	1.135	1.206
University education	<0.001				<0.001			
Not applicable	<0.001	2.156	1.950	2.384	<0.001	1.669	1.530	1.820
Place of residence								
Urban	<0.001				<0.001			
Rural	<0.001	1.270	1.225	1.317	<0.001	1.276	1.231	1.322
Peri-urban	0.156	1.116	0.959	1.298	0.105	1.130	0.975	1.309
Living in a Flu outbreak region	<0.001	0.755	0.739	0.770	<0.001	0.766	0.750	0.781
Received Flu vaccine	0.028	1.021	1.002	1.040	0.018	1.023	1.004	1.042
Constant	<0.001	0.400			<0.001	0.394		

*OR, odds ratio; 95%CI, 95% confidence interval; y.o., years. old.*

*We adopted an alpha error of 0.05. The other causes included SARI due to Influenzae virus infection, SARI due to other respiratory viruses' infection, SARI due to other known etiological agents, and SARI due to SARS-CoV-2 infection (patients with COVID-19).*

a *The statistical analysis included the following patients' features: sex, age, race, educational level, place of residence, residence in a Flu outbreak region, Flu vaccine status during the last vaccination campaign, treatment for SARI symptoms with an antiviral drug, need for intensive care unit, closure criteria, and outcome.*

b *The statistical analysis included the following patients' features: sex, age, race, educational level, place of residence, residence in a Flu outbreak region, and Flu vaccine status during the last vaccination campaign*.

**Table 5:** Multivariate analysis using Binary Logistic Regression to predict the chance of death and the features of the hospitalized patients due to severe acute respiratory infection (SARI) in Brazil during the Coronavirus Disease (COVID)-19 pandemic.

**Patients' features**	***P***	**OR**	**95%CI for OR**	
			**Lower**	**Upper**
Sex (Female)	<0.001	0.815	0.794	0.838
Age				
<1 y.o.	<0.001	0.184	0.135	0.250
1–12 y.o.	<0.001	0.121	0.098	0.149
13–24 y.o.	<0.001	0.479	0.425	0.541
25–60 y.o.	<0.001			
61–72 y.o.	<0.001	2.538	2.450	2.629
73–85 y.o.	<0.001	4.183	4.025	4.347
+85 y.o.	<0.001	7.343	6.986	7.718
Race				
White	<0.001			
Black	<0.001	1.310	1.238	1.387
Asian	0.161	1.100	0.963	1.257
Individuals from a multiracial background	<0.001	1.389	1.349	1.430
Indigenous peoples	<0.001	1.988	1.623	2.436
Educational level				
Illiterate	<0.001	2.191	2.054	2.336
1^st^ fundamental cycle	<0.001	1.901	1.807	2.000
2^nd^ fundamental cycle	<0.001	1.652	1.566	1.743
High school	<0.001	1.365	1.297	1.437
University education	<0.001			
Not applicable	<0.001	2.590	1.970	3.404
Living in a Flu outbreak region	<0.001	1.135	1.100	1.171
Received Flu vaccine	<0.001	0.748	0.727	0.769
Used antiviral drug to treat the clinical signs	<0.001	0.912	0.882	0.944
Need for intensive care unit	<0.001	2.561	2.484	2.640
Mechanical ventilatory support				
Invasive	<0.001	12.151	11.606	12.722
Non-invasive	<0.001	1.865	1.802	1.930
Not required	<0.001			
SARI categorizes				
SARI due to Influenza	0.001	0.541	0.380	0.770
SARI due to other respiratory viruses' infection	<0.001	0.278	0.201	0.384
SARI due to another known etiological agent	<0.001	0.614	0.473	0.799
SARI due to an undefined etiological agent	<0.001	0.463	0.449	0.478
SARI due to COVID-19 (SARS-CoV-2)	<0.001			
Constant	<0.001	0.045		

*SE, standard error; df, degrees of freedom; OR, odds ratio; 95%CI, 95% confidence interval; y.o., years old.*

*We adopted an alpha error of 0.05*.

#### Multivariate Analysis of SARI Patients With an Undefined Etiological Agent vs. Patients With COVID-19

The first model containing the selected markers was significant in differentiating the SARI due to an undefined agent from patients with COVID-19 [X^2^: 31,250.682; *P* < 0.001; Nagelkerke's R^2^: 0.213]. Among the patients' features, the following were significant to classify SARI due to an undefined etiological agent and COVID-19 diagnosis: female (OR: 1.116; 95%CI: 1.093–1.139), age, that is, those aged <1 y.o. (OR: 7.556; 95%CI: 6.557–8.709), those aged between 1 and 12 y.o. (OR: 9.159; 95%CI: 8.448–9.923), 13–24 y.o. (OR: 3.571; 95%CI: 3.379–3.774), 73–85 y.o, (OR: 1.438; 95%CI: 1.392–1.485), and +85 y.o. (OR: 2.091; 95%CI: 2.002–2.183), Black individuals (OR: 1.224; 95%CI: 1.172–1.279), illiterate (OR: 2.186; 95%CI: 2.077–2.301), 1^st^ fundamental cycle (OR: 1.858; 95%CI: 1.788–1.93), 2^nd^ fundamental cycle (OR: 1.605; 95%CI: 1.543–1.671), high school (OR: 1.246; 95%CI: 1.201–1.394), living in a rural area (OR: 1.257; 95%CI: 1.202–1.314), to use an antiviral drug to treat clinical signs (OR: 1.376; 95%CI: 1.341–1.412), clinical – epidemiological closure criterion (OR: 1.277; 95%CI: 1.136–1.434), clinical closure criterion (OR: 4.307; 95%CI: 4.002–4.635), and death not related to SARI (OR: 14.35; 95%CI: 12.595–16.439). All the patients' features and the reference groups were summarized in Table 4.

In the second model, the following patients' features were significant to classify SARI due to an undefined agent and COVID-19 diagnosis [X^2^: 21,229.12; *P* < 0.001; Nagelkerke's R^2^: 0.117]: female (OR: 1.13; 95%CI: 1.11–1.15), age, that is, those aged <1 y.o. (OR: 6.603; 95%CI: 5.833–7.474), those aged between 1 and 12 y.o. (OR: 7.782; 95%CI: 7.265–8.334), 13–24 y.o. (OR: 2.861; 95%CI: 2.736–2.993), 73–85 y.o. (OR: 1.104; 95%CI: 1.076–1.134), and +85 y.o. (OR: 1.438; 95%CI: 1.387–1.49), Black individuals (OR: 1.218; 95%CI: 1.175–1.264), illiterate (OR: 2.018; 95%CI: 1.935–2.104), 1^st^ fundamental cycle (OR: 1.707; 95%CI: 1.654–1.761), 2^nd^ fundamental cycle (OR: 1.426; 95%CI: 1.380–1.474), high school (OR: 1.167; 95%CI: 1.132–1.204), living in a rural area (OR: 1.27; 95%CI: 1.225–1.317), and received Flu vaccine (OR: 1.021; 95%CI: 1.002–1.04). All the patients' features and the reference groups are summarized in Table 4.

#### Multivariate Analysis of Patients With SARI Due to an Undefined Etiological Agent vs. Patients With Other Causes of SARI

The first model containing the selected markers was significant in differentiating the patients with SARI due to an undefined etiological agent from the patients with other causes of SARI [X^2^: 28,125.350; *P* < 0.001; Nagelkerke's R^2^: 0.191]. Among the patients' features, the following were significant to classify SARI due to an undefined etiological agent and other causes of SARI: female (OR: 1.112; 95%CI: 1.089–1.135), age, that is, those aged <1 y.o. (OR: 5.123; 95%CI: 4.557–5.759), those aged between 1 and 12 y.o. (OR: 6.563; 95%CI: 6.113–7.046), 13–24 y.o. (OR: 3.401; 95%CI: 3.221–3.591), 73–85 y.o. (OR: 1.435; 95%CI: 1.39–1.481), and +85 y.o. (OR: 2.07; 95%CI: 1.983–2.161), Black individuals (OR: 1.223; 95%CI: 1.172–1.277), illiterate (OR: 2.161; 95%CI: 2.054–2.273), 1^st^ fundamental cycle (OR: 1.855; 95%CI: 1.786–1.927), 2^nd^ fundamental cycle (OR: 1.606; 95%CI: 1.544–1.671), high school (OR: 1.252; 95%CI: 1.206–1.299), living in a rural area (OR: 1.261; 95%CI: 1.207–1.318), to use antiviral drug to treat clinical signs (OR: 1.312; 95%CI: 1.28–1.346), clinical – epidemiological closure criterion (OR: 1.307; 95%CI: 1.165–1.467), clinical closure criterion (OR: 4.252; 95%CI: 3.956–4.57), and death not related to SARI (OR: 12.546; 95%CI: 11.099–14.182). We summarized the patients' features and the reference groups in Table 4.

In the second model, the following patients' features were significant to classify SARI with an undefined etiological agent and other causes of SARI [X^2^: 18,271.422; *P* < 0.001; Nagelkerke's R^2^: 0.100]: female (OR: 1.126; 95%CI: 1.107–1.146), age, that is, those aged <1 y.o. (OR: 4.766; 95%CI: 4.295–5.289), those aged between 1 and 12 y.o. (OR: 6.007; 95%CI: 5.646–6.39), 13–24 y.o. (OR: 2.773; 95%CI: 2.652–2.899), 73–85 y.o. (OR: 1.109; 95%CI: 1.08–1.139), and +85 y.o. (OR: 1.438; 95%CI: 1.388–1.491), Black individuals (OR: 1.217; 95%CI: 1.174–1.262), illiterate (OR: 1.997; 95%CI: 1.915–2.082), 1^st^ fundamental cycle (OR: 1.702; 95%CI: 1.649–1.756), 2^nd^ fundamental cycle (OR: 1.425; 95%CI: 1.379–1.473), high school (OR: 1.17; 95%CI: 1.135–1.206), living in a rural area (OR: 1.276; 95%CI: 1.231–1.322), and received Flu vaccine (OR: 1.023; 95%CI: 1.004–1.042). All the patients' features and the reference groups are summarized in Table 4.

#### Multivariate Analysis to Predict the Chance of Death in Hospitalized Patients Due to SARI

The model containing the selected markers was significant in predicting the death in hospitalized patients with SARI in Brazil [X^2^: 57,779.281; *P* < 0.001; Nagelkerke's R^2^: 0.41]. Among the patients' features, the following were significant in predicting death among hospitalized patients due to SARI: age, those with 61–72 y.o. (OR: 2.53; 95%CI: 2.45–2.629), 73–85 y.o. (OR: 4.183; 95%CI: 4.025–4.347), and +85 y.o. (OR: 7.343; 95%CI: 6.989–7.718), Black individuals (OR: 1.31; 95%CI: 1.238–1.387), individuals with a multiracial background (OR: 1.389; 95%CI: 1.349–1.43), Indigenous peoples (OR: 1.988; 95%CI: 1.623–2.436), illiterate (OR: 2.191; 95%CI: 2.054–2.336), 1^st^ fundamental cycle (OR: 1.901; 95%CI: 1.807–2), 2^nd^ fundamental cycle (OR: 1.652; 95%CI: 1.566–1.743), high school (OR: 1.365; 95%CI: 1.297–1.437), living in a flu outbreak region (OR: 1.135; 95%CI: 1.1–1.171), need for intensive care unit (OR: 2.561; 95%CI: 2.484–2.64), need for invasive mechanical ventilatory support (OR: 12.151; 95%CI: 11.606–12.722), and non-invasive mechanical ventilatory support (OR: 1.865; 95%CI: 1.802–1.93). Several patients' features [e.g., female sex, younger age, patients who received a flu shot, the use of antiviral drugs, SARI due to Influenza, SARI due to other respiratory infections, SARI due to OEAs (known), and SARI due to an undefined agent] were more frequent in the group of patients who recovered. We summarized the patients' features and the reference groups in Table 5.

### Association Between Features of the Hospitalized Patients Due to SARI in Brazil During the COVID-19 Pandemic According to the Year of Data Collection

In our study, we compared the patients' features according to the year of data collection for two study populations: (i) first analysis: all patients enrolled in the study; (ii) second analysis: only patients with a positive result in the SARS-CoV-2 RT-PCR. For the first analysis, we observed in 2020 a higher proportion of female patients (OR: 1.032; 95%CI: 1.027–1.037) than males; younger [ <1 y.o. (OR: 1.025; 95%CI: 1.008–1.042), 1–12 y.o. (OR: 1.375; 95%CI: 1.359–1.392), and 13–24 y.o. (OR: 1.634; 95%CI: 1.61–1.658)] and older [61–72 y.o. (OR: 1.294; 95%CI: 1.286–1.302), 73–85 y.o. (OR: 1.547; 95%CI: 1.537–1.558), and +85 y.o. (OR: 1.757; 95%CI: 1.74–1.774) patients when compared to patients aged between 25 and 60 y.o.; Black (OR: 1.281; 95%CI: 1.266–1.297), Asian (OR: 1.261; 95%CI: 1.23–1.292), individuals with multiracial background (OR: 1.084; 95%CI: 1.078–1.09), and Indigenous peoples (OR: 1.928; 95%CI: 1.829–2.033) when compared with White ones; low educational level [illiterate (OR: 1.12; 95%CI: 1.1–1.14) and 1^st^ fundamental cycle (OR: 1.052; 95%CI: 1.038–1.067)]; and deaths not relate to SARI (OR: 1.139; 95%CI: 1.114–1.165) when compared to 2021 year. Also, we described a higher proportion of patients who received the flu vaccine in the last Brazilian campaign (OR: 1.971; 95%CI: 1.954–1.988), and that used an antiviral drug to treat the clinical signs (OR: 6.72; 95%CI: 6.646–6.795) in 2020 than 2021. In addition, the patients selected from 2020 were more prone to be classified as SARI due to Influenza (OR: 3.965; 95%CI: 3.688–4.262), SARI due to OEAs (OR: 1.406; 95%CI: 1.34–1.474), and SARI due to an undefined etiological agent (OR: 1.901; 95%CI: 1.891–1.911) (Table 6) when compared to patient selected from 2021.

**Table 6:** Association between features of the hospitalized patients due to severe acute respiratory infection (SARI) in Brazil during the Coronavirus Disease (COVID)-19 pandemic according to the collection period.

**Patient's features**	**Category**	2020^*^	2021^*^	**Total**	***P***	**OR**	**95%CI**
Sex	Female	524,388 (45.9%)	720,728 (45.1%)	1,245,116 (45.4%)	<0.001	1.032	1.027–1.037
	Male	617,832 (54.1%)	876,584 (54.9%)	1,494,416 (54.6%)		1	Reference
Age	<1 y.o.	23,048 (2.0%)	38,238 (2.4%)	61,286 (2.2%)	0.004	1.025	1.008–1.042
	1–12 y.o.	50,992 (4.5%)	63,022 (3.9%)	114,014 (4.2%)	<0.001	1.375	1.359–1.392
	13–24 y.o.	36,796 (3.2%)	38,280 (2.4%)	75,076 (2.7%)	<0.001	1.634	1.610–1.658
	25–60 y.o.	470,160 (41.1%)	799,238 (50.0%)	1,269,398 (46.3%)		1	Reference
	61–72 y.o.	254,149 (22.2%)	333,848 (20.9%)	587,997 (21.5%)	<0.001	1.294	1.286–1.302
	73–85 y.o.	210,092 (18.4%)	230,802 (14.4%)	440,894 (16.1%)	<0.001	1.547	1.537–1.558
	+85 y.o.	97,382 (8.5%)	94,225 (5.9%)	191,607 (7.0%)	<0.001	1.757	1.740–1.774
Race	White	434,573 (48.2%)	670,550 (50.9%)	1,105,123 (49.8%)		1	Reference
	Black	56,032 (6.2%)	67,472 (5.1%)	123,504 (5.6%)	<0.001	1.281	1.266–1.297
	Asian	12,057 (1.3%)	14,757 (1.1%)	26,814 (1.2%)	<0.001	1.261	1.230–1.292
	Individuals from a multiracial background	395,041 (43.9%)	562,456 (42.7%)	957,497 (43.2%)	<0.001	1.084	1.078–1.090
	Indigenous peoples	3,100 (0.3%)	2,481 (0.2%)	5,581 (0.3%)	<0.001	1.928	1.829–2.033
Educational level	Illiterate	36,341 (8.5%)	42,377 (7.5%)	78,718 (7.9%)	<0.001	1.120	1.100–1.140
	1^st^ fundamental cycle	115,419 (27.0%)	143,260 (25.5%)	258,679 (26.1%)	<0.001	1.052	1.038–1.067
	2^nd^ fundamental cycle	72,134 (16.8%)	98,604 (17.5%)	170,738 (17.2%)	<0.001	0.955	0.941–0.970
	High school	115,525 (27.0%)	169,239 (30.1%)	284,764 (28.7%)	<0.001	0.892	0.880–0.904
	University education	54,593 (12.8%)	71,298 (12.7%)	125,891 (12.7%)		1	Reference
	Not applicable	34,101 (8.0%)	37,700 (6.7%)	71,801 (7.2%)		-	-
Place of residence	Urban	959,510 (94.6%)	1,311,499 (94.1%)	2,271,009 (94.3%)		1	Reference
	Rural	50,545 (5.0%)	76,286 (5.5%)	126,831 (5.3%)	<0.001	0.906	0.895–0.916
	Peri-urban	4,027 (0.4%)	6,025 (0.4%)	10,052 (0.4%)	<0.001	0.914	0.878–0.951
Living in a Flu outbreak region	Yes	207,673 (28.0%)	53,796 (30.9%)	261,469 (28.5%)	<0.001	0.867	0.857–0.877
	No	535,181 (72.0%)	120,204 (69.1%)	655,385 (71.5%)		1	Reference
Received Flu vaccine	Yes	153,446 (32.7%)	127,214 (19.8%)	280,660 (25.2%)	<0.001	1.971	1.954–1.988
	No	315,497 (67.3%)	515,554 (80.2%)	831,051 (74.8%)		1	Reference
Used antiviral drug to treat the clinical signs	Yes	180,826 (21.7%)	42,218 (4.0%)	223,044 (11.8%)	<0.001	6.720	6.646–6.795
	No	650,953 (78.3%)	1,021,342 (96.0%)	1,672,295 (88.2%)		1	Reference
Intensive care unit	Yes	328,149 (34.3%)	464,605 (34.6%)	792,754 (34.5%)	<0.001	0.987	0.982–0.993
	No	627,365 (65.7%)	876,840 (65.4%)	1,504,205 (65.5%)		1	Reference
Mechanical ventilatory support	Invasive	167,340 (17.8%)	253,464 (19.1%)	420,804 (18.5%)	<0.001	0.576	0.572–0.581
	Non-Invasive	474,813 (50.4%)	813,942 (61.2%)	1,288,755 (56.7%)	<0.001	0.509	0.506–0.513
	Not required	300,128 (31.9%)	261,989 (19.7%)	562,117 (24.7%)		1	Reference
Closure criterion	Laboratorial criterion	981,259 (92.1%)	1,249,557 (89.1%)	2,230,816 (90.4%)	<0.001	1	Reference
	Clinical - Epidemiological	10,967 (1.0%)	21,405 (1.5%)	32,372 (1.3%)	<0.001	0.652	0.638–0.678
	Clinical	46,582 (4.4%)	54,802 (3.9%)	101,384 (4.1%)	<0.001	1.082	1.069–1.096
	Clinical - Image exams	27,148 (2.5%)	76,110 (5.4%)	103,258 (4.2%)	<0.001	0.454	0.448–0.461
Outcome	Cure	703,085 (69.6%)	874,194 (67.3%)	1,577,279 (68.3%)		1	Reference
	Death	293,037 (29.0%)	408,570 (31.5%)	701,607 (30.4%)	<0.001	0.892	0.887–0.897
	Death not related to SARI	14,608 (1.4%)	15,943 (1.2%)	30,551 (1.3%)	<0.001	1.139	1.114–1.165
SARI categories	SARI due to Influenza	2,416 (0.2%)	1,058 (0.1%)	3,474 (0.1%)	<0.001	3.965	3.688–4.262
	SARI due to other respiratory viruses' infection	4,648 (0.4%)	11,979 (0.7%)	16,627 (0.6%)	<0.001	0.674	0.651–0.697
	SARI due to another known etiological agent	3,072 (0.3%)	3,794 (0.2%)	6,866 (0.3%)	<0.001	1.406	1.340–1.474
	SARI due to an undefined etiological agent	468,407 (41.0%)	427,800 (26.8%)	896,207 (32.7%)	<0.001	1.901	1.891–1.911
	SARI due to COVID-19 (SARS-CoV-2)	664,076 (58.1%)	1,153,022 (72.2%)	1,817,098 (66.3%)		1	Reference

*We presented the data as the number of individuals (N) and percentage (%).*

*We did the statistical analyses using the chi-square test. We adopted an alpha error of 0.05.*

*SARS-CoV-2, Severe Acute Respiratory Syndrome Coronavirus 2; OR, odds ratio; 95%CI, 95% confidence interval; y.o., years old.*

* *We further divided the patients into two periods. The first period was from December 29, 2019, to December 31, 2020*.

We also compared the patients' features according to the year of data collection for patients with a positive result in the SARS-CoV-2 RT-PCR, and we observed in 2020 a higher proportion of female patients (OR: 1.007; 95%CI: 1.001–1.013) when compared to males; younger [ <1 y.o. (OR: 1.706; 95%CI: 1.635–1.78), 1–12 y.o. (OR: 1.893; 95%CI: 1.833–1.955), and 13–24 y.o. (OR: 1.447; 95%CI: 1.416–1.479)] and older [61–72 y.o. (OR: 1.359; 95%CI: 1.348–1.369), 73–85 y.o. (OR: 1.632; 95%CI: 1.618–1.646), and +85 y.o. (OR: 1.828; 95%CI: 1.805–1.851)] patients when compared to patients aged between 25 and 60 y.o.; Black (OR: 1.354; 95%CI: 1.334–1.375), Asian (OR: 1.367; 95%CI: 1.326–1.409), individuals with multiracial background (OR: 1.191; 95%CI: 1.182–1.199), and Indigenous peoples (OR: 2.706; 95%CI: 2.532–2.892) when compared with White ones; low educational level [illiterate (OR: 1.183; 95%CI: 1.156–1.211)]; and deaths not related to SARI (OR: 1.471; 95%CI: 1.381–1.566) when compared to 2021. Also, we described a higher proportion of patients who received the flu vaccine in the last Brazilian campaign (OR: 2.048; 95%CI: 2.026–2.07), and that used an antiviral drug to treat the clinical signs (OR: 6.965; 95%CI: 6.871–7.061) in 2020 than 2021. The data was summarized in the Table 7.

**Table 7:** Association between features of the hospitalized patients due to severe acute respiratory infection (SARI) in Brazil due to the Coronavirus Disease (COVID)-19 according to the data collection period.

**Patient's features**	**Category**	2020^*^	2021^*^	**Total**	***P***	**OR**	**95%CI**
Sex	Female	294,656 (44.4%)	509,747 (44.2%)	804,403 (44.3%)	0.031	1.007	1.001–1.013
	Male	369,280 (55.6%)	643,127 (55.8%)	1,012,407 (55.7%)		1	Reference
Age	<1 y.o.	3,876 (0.6%)	4,846 (0.4%)	8,722 (0.5%)	<0.001	1.706	1.635–1.780
	1–12 y.o.	7,178 (1.1%)	8,087 (0.7%)	15,265 (0.8%)	<0.001	1.893	1.833–1.955
	13–24 y.o.	14,325 (2.2%)	21,109 (1.8%)	35,434 (2.0%)	<0.001	1.447	1.416–1.479
	25–60 y.o.	301,199 (45.4%)	642,332 (55.7%)	943,531 (51.9%)		1	Reference
	61–72 y.o.	162,728 (24.5%)	255,418 (14.0%)	418,146 (23.0%)	<0.001	1.359	1.348–1.369
	73–85 y.o.	123,531 (18.6%)	161,455 (14.0%)	284,986 (15.7%)	<0.001	1.632	1.618–1.646
	+85 y.o.	51,239 (7.7%)	59,775 (5.2%)	111,014 (6.1%)	<0.001	1.828	1.805–1.851
Race	White	250,127 (48.2%)	508,116 (53.1%)	758,243 (51.4%)		1	Reference
	Black	31,017 (6.0%)	46,525 (4.9%)	77,542 (5.3%)	<0.001	1.354	1.334–1.375
	Asian	7,249 (1.4%)	10,775 (1.1%)	18,024 (1.2%)	<0.001	1.367	1.326–1.409
	Individuals from a multiracial background	228,083 (44.0%)	389,155 (40.7%)	617,238 (41.9%)	<0.001	1.191	1.182–1.199
	Indigenous peoples	2,042 (0.4%)	1,533 (0.2%)	3,575 (0.2%)	<0.001	2.706	2.532–2.892
Educational level	Illiterate	17,306 (7.1%)	23,402 (5.7%)	40,708 (6.2%)	<0.001	1.183	1.156–1.211
	1^st^ fundamental cycle	63,179 (26.0%)	105,848 (25.7%)	169,027 (25.8%)	<0.001	0.955	0.940–0.971
	2^nd^ fundamental cycle	43,510 (17.9%)	77,456 (18.8%)	120,966 (18.5%)	<0.001	0.899	0.883–0.915
	High school	75,409 (31.0%)	139,782 (33.9%)	215,191 (32.8%)	<0.001	0.863	0.850–0.877
	University education	38,398 (15.8%)	61,437 (14.9%)	99,835 (15.2%)		1	Reference
	Not applicable	5,063 (2.1%)	4,525 (1.1%)	9,588 (1.5%)		-	-
Place of residence	Urban	559,656 (95.2%)	957,366 (94.4%)	1,517,022 (94.7%)		1	Reference
	Rural	25,934 (4.4%)	52,922 (5.2%)	78,856 (4.9%)	<0.001	0.838	0.826–0.851
	Peri-urban	2,120 (0.4%)	3,507 (0.3%)	5,627 (0.4%)	0.224	1.034	0.980–1.091
Living in a Flu outbreak region	Yes	129,409 (30.5%)	38,983 (31.3%)	168,392 (30.7%)	<0.001	0.961	0.948–0.974
	No	295,062 (69.5%)	85,420 (68.7%)	380,482 (69.3%)		1	Reference
Received Flu vaccine	Yes	85,822 (32.4%)	91,903 (19.0%)	177,725 (23.7%)	<0.001	2.048	2.026–2.070
	No	179,115 (67.6%)	392,790 (81.0%)	571,905 (76.3%)		1	Reference
Used antiviral drug to treat the clinical signs	Yes	100,925 (21.2%)	29,033 (3.7%)	129,958 (10.3%)	<0.001	6.965	6.871–7.061
	No	375,069 (78.8%)	751,508 (96.3%)	1,126,577 (89.7%)		1	Reference
Intensive care unit	Yes	209,877 (37.5%)	375,902 (37.5%)	585,779 (37.5%)	0.597	0.998	0.992–1.005
	No	349,909 (62.5%)	625,565 (62.5%)	975,474 (62.5%)		1	Reference
Mechanical ventilatory support	Invasive	108,760 (19.7%)	212,540 (21.4%)	321,300 (20.8%)	<0.001	0.520	0.515–0.525
	Non-Invasive	287,920 (52.1%)	620,228 (62.6%)	908,148 (58.8%)	<0.001	0.472	0.468–0.476
	Not required	155,636 (28.2%)	158,109 (16.0%)	313,745 (20.3%)		1	Reference
Closure criterion	Laboratorial criterion	606,937 (93.1%)	1,000,651 (89.7%)	1,607,588 (90.9%)		1	Reference
	Clinical - Epidemiological	5,626 (0.9%)	13,471 (1.2%)	19,097 (1.1%)	<0.001	0.689	0.667–0.710
	Clinical	14,350 (2.2%)	29,154 (2.6%)	43,504 (2.5%)	<0.001	0.812	0.795–0.828
	Clinical - Image exams	25,054 (3.8%)	72,575 (6.5%)	97,629 (5.5%)	<0.001	0.569	0.561–0.578
Outcome	Cure	405,324 (65.5%)	672,546 (64.8%)	1,077,870 (65.1%)		1	Reference
	Death	211,873 (34.2%)	363,014 (35.0%)	574,887 (34.7%)	<0.001	0.968	0.962–0.975
	Death not related to SARI	1,850 (0.3%)	2,087 (0.2%)	3,937 (0.2%)	<0.001	1.471	1.381–1.566

*We presented the data as the number of individuals (N) and percentage (%).*

*We did the statistical analyses using the chi-square test. We adopted an alpha error of 0.05.*

*SARS-CoV-2, Severe Acute Respiratory Syndrome Coronavirus 2; OR, odds ratio; 95%CI, 95% confidence interval; y.o., years old.*

* *We further divided the patients into two periods. The first period was from December 29, 2019, to December 31, 2020*.

## Discussion

To the best of our knowledge, this study was the first one to evaluate and compare the epidemiological features of five distinct groups, namely individuals with SARI due to Influenza virus infection, SARI due to other respiratory viruses' infection, SARI due to OEAs (known), SARI due to COVID-19 and SARI due to an undefined etiological agent, in Brazil and worldwide. Interestingly, our results showed that several epidemiological features were associated with an increased risk of being diagnosed with SARI due to an undefined etiological agent, which might be due to, at least in part, the poor testing policy for SARS-CoV-2 in Brazil. Brazil only accounts for 304.64 tests per 1,000 inhabitants, behind countries such as the USA (1,989.7 tests per 1,000 inhabitants), Argentina (571.21 tests per 1,000 inhabitants), and Chile (1,319.51 per 1,000 inhabitants) (Ritchie et al., 2020). Brazil faces an intense underreporting of COVID-19 cases and deaths, as reported in the literature (do Prado et al., 2020; Veiga e Silva et al., 2020; Albani et al., 2021; Carvalho et al., 2021). Interestingly, a study showed an increase in the notifications of SARI death, ranging from 553 to 6,991%, in several Brazilian capitals in 2020, when compared to previous years (Veiga e Silva et al., 2020). In this context, most of the deaths notified as SARI only might be named as COVID-19 ones as death causes.

The authors did not wholly elucidate the mechanisms associated with “preference” for the male sex of the SARS-CoV-2, and the literature demonstrated conflicting results. However, the male sex seems to be at increased death and need for intensive care unit (Grasselli et al., 2020; Gupta S. et al., 2020; Peckham et al., 2020; Jun et al., 2021; Pijls et al., 2021), perhaps due to underlying comorbidities, hormonal factors, or even differences between immune systems (La Vignera et al., 2020; Maleki Dana et al., 2020; Scully et al., 2020; Sharma et al., 2020; Jun et al., 2021). Also, women are more worried about their health and visit health care units more frequently (NCHS Pressroom, 2019; PNS), especially in Brazil, in which 82.3% of the women had at least one consult with a doctor per year, while only 69.4% of the men had a consult (PNS). In our study, the female sex had an enhanced chance of being diagnosed with SARI with an undefined etiological agent; perhaps this might be due to low SARS-CoV-2 testing in this sex, which might have led to testing more of those at higher risk of death, which as the male sex.

Regarding age, the prevalence of SARS-CoV-2 in children was lower when compared to adults, and most of the infected children did not have severe symptoms, only those common in the regular cold (CDC COVID-19 Response Team, 2020; Dong et al., 2020; Wu and McGoogan, 2020; Borrelli et al., 2021; Viner et al., 2021). In Brazil, we can attribute the low number of COVID-19 diagnoses among children to the low number of RT-PCR SARS-CoV-2 tests performed, which we can explain by the low prevalence of SARS-CoV-2 and the less severe cases in these age groups (CDC COVID-19 Response Team, 2020; Dong et al., 2020; Wu and McGoogan, 2020; Borrelli et al., 2021; Viner et al., 2021), which might contribute to the diagnosis of SARI to the undefined cause. Even though we face a pandemic, there is over 200 virus, other than SARS-CoV-2, which can cause diseases related to the respiratory system (Berman et al., 1983). The most common viruses are Respiratory Syncytial Virus, Parainfluenza, Adenovirus, Influenza, Enterovirus, Human Metapneumovirus, and Rhinovirus (Hazlett et al., 1988; Ruutu et al., 1990; Ray et al., 1993; Shi et al., 2015). In Brazil, the prevalence of these viruses in children is also elevated, especially in those under five y.o. (Tsuchiya et al., 2005; Thomazelli et al., 2007). Perhaps, this might have contributed to our findings, in which the younger individuals have an enhanced chance of being diagnosed with SARI due to an undefined etiological agent. Since the pandemic's beginning, several studies showed that older age, especially that of +70 y.o., is a risk factor for worst outcomes, such as death, need for intensive care unit, and need for mechanical ventilatory support (Liu et al., 2020; Richardson et al., 2020; Shen et al., 2020; Wei et al., 2020; Pijls et al., 2021). Curiously, in our study, those older than 73 y.o. also increased the chance of SARI due to an undefined etiological agent and not SARI due to COVID-19.

The situation brought by COVID-19 affects those with underlying conditions such as cardiovascular disease or obesity (Williamson et al., 2020; Yoshikawa and Asaba, 2021) and low socioeconomic status and educational level (Abedi et al., 2020; Hawkins et al., 2020; Niedzwiedz et al., 2020; Ribeiro et al., 2021; Yoshikawa and Asaba, 2021). For example, São Paulo city had four times more mortality due to COVID-19 in areas with a low percentage of individuals with a university degree than in areas with a high rate of individuals with a university degree (Ribeiro et al., 2021). The race also plays a vital role in the outcomes related to COVID-19 since individuals from neglected races, like Black, Indigenous peoples, and individuals with multiracial backgrounds, were more affected by the disease, as shown in the literature and our data (Baqui et al., 2020; Martins-Filho et al., 2021; Mendes et al., 2021).

The Brazilian Unified Health System (*Sistema Único de Saúde*, SUS) has three principles: universality, equity, and integrality (Princípios do SUS). Unfortunately, the access to SUS by its population is full of inequalities between distinct social and races groups (Lima-Costa et al., 2002; Travassos et al., 2006; Stopa et al., 2017), as observed in the Brazilian National Health Survey from 2013, in which individuals with enhanced educational levels had greater access to Health System compared to those with low educational levels (Stopa et al., 2017). In the same way, individuals living in the South or Southeast regions had greater access to the health system, perhaps due to life in these regions having better social status, enhanced growth domestic product, improved urbanization rate, and more health investment (IBGE; Saúde - Portal da transparência). In addition, the South and Southeast regions had the highest Human Development Index in Brazil, and most of the population from these regions live in cities where access to the SUS is easier (Cazelli et al., 2002; Oliveira and Dallabrida, 2013; Stopa et al., 2017; Azevedo). On top of that, individuals from neglected races, especially the Black and the Indigenous peoples, have classically low access to health care centers in Brazil due to structural racism, the difficulties in reaching a basic health unit since most of these people live in rural areas (Silva et al., 2020; Mendes et al., 2021).

In our study, the lowest educational level, Black individuals, and individuals living in rural areas were at increased risk of being categorized as SARI due to an undefined etiological agent. Perhaps, these individuals had low access to health care centers to perform the SARS-CoV-2 RT-PCR (Rentsch et al., 2020; Silva et al., 2020; Mody et al., 2021; Souch and Cossman, 2021). Noteworthy, in individuals of the Black race or living in rural areas, the COVID-19 testing was less performed (Rentsch et al., 2020; Mody et al., 2021; Souch and Cossman, 2021), which might have also contributed to our results. Curiously in our data, the Indigenous peoples were more prone to be diagnosed with SARI due to COVID-19 and not SARI with an undefined etiological agent, as we expected. Our data regarding Indigenous peoples presented results as the previous studies that showed an increased burden of COVID-19 in these peoples (Cupertino et al., 2020; Palamim et al., 2020; Santos et al., 2020; Mendes et al., 2021; Sansone et al., 2022). The increased diagnosis rate of COVID-19 might be partly due to the law n°14.021/2020, which reinforces the right of Indigenous peoples to have RT-PCR SARS-CoV-2 tests or other tools that can identify COVID-19 in their territory (Nacional).

Unfortunately, few studies tried to evaluate the epidemiologic characteristics differences between SARI and COVID-19. For example, a study conducted in North India enrolled 212 SARI patients, in which the authors observed patients with COVID-19 are older than individuals with SARI. However, the clinical presentation was similar in both groups (Pannu et al., 2021). Another study conducted in India (Sharma et al., 2021) enrolled 500 participants, in which only 88 were positive for COVID-19. Similar to the study conducted in North India, the authors observed a similar clinical profile between individuals with COVID-19 and SARI, and also, older patients were diagnosed with COVID-19 (Sharma et al., 2021). Finally, the most extensive study in India accounted for nearly 5,000 individuals with SARI, out of which 104 were diagnosed with COVID-19 (Gupta, N. et al., 2020), being the COVID-19 group had an increased number of male patients. To the best of our knowledge, no study had the groups proposed by our study, which makes comparison difficult; however, in our study, individuals from the COVID-19 group were also older and male.

In Brazil, only two other studies tried to compare individuals with SARI and COVID-19 (Bastos et al., 2020; Niquini et al., 2020). The study performed by Niquini et al. (2020) accounted for 39,349 hospitalized patients with SARI up to the 21^st^ epidemiologic week of 2020. The patients were further divided into a COVID-19 and a SARI due to Influenza, being that most of the patients had COVID-19. Similar to our study, the COVID-19 group accounted for more White patients (Niquini et al., 2020). Another study performed by Bastos et al. (2020) evaluated the notification of SARI until the 12^th^ week of 2020. The authors observed enhanced hospitalization of SARI in 2020 when compared to previous years. In fact, for the 12^th^ epidemiologic week, the hospitalizations seem to have increased 59% from previous years. A higher hospitalization rate of older individuals was also noted (Bastos et al., 2020), which is in accordance with our study.

The common cold and COVID-19, even though caused by different etiological agents, share some clinical similarities, as both can manifest myalgia, fever, cough, and headache (Czubak et al., 2021). In our results, people living in a flu outbreak region might test for COVID-19 more often since the clinical presentation is similar. Unfortunately, individuals with SARI, due to an undefined etiological agent, received more antiviral drugs (e.g., oseltamivir and zanamivir) to treat the clinical symptoms. The antiviral drugs were first developed to treat Influenza (Jefferson et al., 2014), and their use in COVID-19 is not approved yet, since some clinical studies are still in progress (University Hospitals Coventry and Warwickshire NHS Trust, 2020; Elkholy, 2021). A higher proportion of individuals received antiviral drugs in 2020 when compared to 2021; perhaps due to the fact in 2020, there were more individuals with SARI due to an undefined etiological agent, which might have led the physicians to treat individuals as it was caused by Influenza virus.

Brazil accounts for a significant number of COVID-19 underreporting. In this context, many patients categorized as SARI due to an undefined etiological agent are patients with COVID-19 (Marson and Ortega, 2020; Veiga e Silva et al., 2020; Carvalho et al., 2021; Kupek, 2021). In fact, all the Brazilian states account for at least some underreporting of COVID-19 cases and deaths (Paixão et al., 2021); however, not homogenously. It seems the states with the highest underreporting of cases were Minas Gerais and Mato Grosso, which might be explained by different social, economic, and demographic characteristics, being unique in each state (Paixão et al., 2021). Some singularities across the Brazilian states, including socioeconomic characteristics, and COVID-19 testing policies, might have played a crucial role in the underreports. Although the testing policies across the states might be similar, they harbor some differences, which could explain, at least in part, the different underreports rates (Boschiero et al., 2021a,b; Cidadão - Secretaria da Saúde - Governo do Estado de São Paulo; Secretaria de Estado de Saúde de Minas Gerais).

Furthermore, since we included the beginning of the COVID-19 pandemic in our analysis, the first health public policies might have affected our results. For instance, the Federal Government only released a “National Plan for Expansion of COVID-19 testing” at the end of 2021; in this scenario, the absence of a public health policy aimed at COVID-19 diagnosis at the beginning of the pandemic can be associated with a higher possible underdiagnosis, which reflects the higher number of patients classified as individuals with an undefined etiological agent (Ministério da Saúde lança Plano Nacional de Expansão da Testagem para Covid-19). Also, at the beginning of the pandemic, Brazil performed more antibody tests than RT-PCR tests for COVID-19, which could decrease diagnosis accuracy since the RT-PCR is the gold standard (Oliveira et al., 2020; Boschiero et al., 2021a,b; Localiza SUS). Brazil also accounted for at least two COVID-19 waves (Zeiser et al., 2022), with differences across them; the second wave might even be more severe than the first, with more deaths and hospitalizations (Bastos et al., 2021; Zeiser et al., 2022). In our study, we also observed a more severe clinical profile in individuals from 2021 since more of them required ventilatory support, both invasive and non-invasive, and more individuals died when compared to 2020. One of the main reasons different outcomes and clinical characteristics were observed between the waves might be due to different SARS-CoV-2 variants. In the first wave, which occurred mainly during 2020, the predominant variant was B.1.1.33 and B.1.1.28. In the second wave, which occurred mainly during 2021, a higher prevalence of the Gamma (P.1) variant was observed (Zeiser et al., 2022). Curiously, the emergence of the Gamma variant coincided with the collapse of the healthcare system in Manaus (Boschiero et al., 2021a,b). Furthermore, the Delta variant was also important, as from July 2021 until 2022, it quickly became one of the most prevalent variants in Brazil and might even be more transmissible (Planas et al., 2021; Giovanetti et al., 2022).

Two recent Brazilian studies also observed an enhancement in severe cases of COVID-19 and also enhanced mortality, especially among the youngest women, which corresponded to the emergence of the Gamma variant (Freitas et al., 2021; Banho et al., 2022). Another study also pointed out the Gamma variant might be nearly two times more transmissible than non-Gamma variants (Coutinho et al., 2021). In our study, a higher proportion of SARI due to COVID-19 was observed in 2021 when compared to 2020 (58.1% vs. 72.2%). However, several other reasons might also have played a key role in the diagnosis of SARI due to COVID-19 such as an improvement in the diagnosis policies, widespread testing and the higher investment in RT-PCR tests instead antibodies tests (Boschiero et al., 2021a,b; Ministério da Saúde lança Plano Nacional de Expansão da Testagem para Covid-19), and the rise of new and more transmissible variants might have also been important in the increased of SARI due to COVID-19. In fact, even though Brazil started the COVID-19 vaccination in 2021 (Boschiero et al., 2021a), more severe cases were reported in 2021 (Freitas et al., 2021), despite the vaccination, which reinforces the hypothesis the Gamma lineage is more pathogenic.

Regarding the Flu vaccine, studies have shown an inverse correlation between the Flu vaccine coverage and the worst outcomes of the SARS-CoV-2 infection, such as a higher risk for the need for hospitalization, need for intensive care units, and death (Amato et al., 2020; Fink et al., 2021; Reina, 2021). The vaccine affects innate immunity, determining an overall (non-target) protective effect that would affect viruses not initially contained in the vaccine by trained immunity. The Flu vaccine can induce non-specific immunotherapeutic mechanisms that increase the immune response to other pathogens, such as the SARS-CoV-2 (Netea et al., 2020; Fink et al., 2021). Curiously, we observed that vaccinated individuals against Flu were less prone to be diagnosed with SARI by an undefined etiological agent than COVID-19. We also noticed that fewer individuals were vaccinated against Flu in 2021 when compared to 2020; perhaps individuals might have been scared to go outside since Brazil was facing one of the highest numbers of deaths due to COVID-19 at that time (Boschiero et al., 2021a).

Interestingly, the necessity of intensive care unit treatment for individuals with SARI, especially those caused by Influenza and SARI due to COVID-19, is similar. Nearly 10–30% of patients needed intensive care unit treatment (Booth et al., 2003; Choi et al., 2003; Fowler et al., 2003; Lee et al., 2003; Lew et al., 2003; Peiris et al., 2003; Beumer et al., 2019; Guan et al., 2020; Phua et al., 2020; Zangrillo et al., 2020; Hajjar et al., 2021). However, in our study, those with SARI due to an undefined etiological agent were less prone to need intensive care unit treatment than patients with COVID-19. Since Brazil faced a major COVID-19 outbreak, with the health system's collapse and almost 100% occupancy in Brazilian intensive care units (Buss et al., 2021; Naveca et al., 2021; Sabino et al., 2021; Silva and Pena, 2021), perhaps the attending doctors reserved the few remaining intensive care unit beds for those with confirmed COVID-19 diagnoses. Furthermore, we observed the mortality of those with SARI due to an undefined etiological agent to be lower if compared to SARI due to COVID-19, which is similar to the literature, in which the hospitalized patients with COVID-19 accounted for nearly 37% of lethality rate and hospitalized Influenza patients for only 6.7% of lethality rate (Rößler et al., 2021; Zeiser et al., 2022). Importantly, in Brazil, the severe SARI cases were more tested than other patients (Boschiero et al., 2021a,b), which could be associated with a low number of deaths among the patients with SARI due to an undefined etiological agent.

The literature described a higher incidence of venous thromboembolism in patients infected by the SARS-CoV-2, and nearly one-third of patients have thrombotic events. Several thrombotic mechanisms are essential in the prothrombotic pathophysiology of COVID-19, such as immobilization, endothelial dysfunction, decreased fibrinolysis, and increased coagulation (Obi et al., 2019; Giannis et al., 2020; Helms et al., 2020; Loo et al., 2021). In a multicenter study, nearly 42% of the patients with COVID-19 had thrombotic events, with the most common being pulmonary embolism (Helms et al., 2020). The thrombotic events might contribute to our data, in which patients with COVID-19 had an increased chance of dying. However, we described more deaths unrelated to SARI for patients grouped as SARI due to another OEA (known) or an undefined etiological agent. The SIVEP-Flu did not explain which kind of death occurred in the dataset. However, we can postulate that some patients who presented deaths associated with lung disease progression or secondary outcomes, such as venous thromboembolism, were classified in the wrong groups and, in this case, could also be patients with COVID-19.

We hypothesize that many patients were categorized as having an undetermined etiological agent to be, in fact, COVID-19. However, we are unsure since we do not have a positive test confirming COVID-19. Besides that, many of these patients have epidemiology characteristics, such as younger age, Black race, lower education level, and living in a rural area might have contributed to lower COVID-19 testing, as aforementioned. In addition, even though these patients were not tested, the pretest probability of having COVID-19 is high, and they might not even know they are infected, as observed by a previous Brazilian study (Hallal et al., 2020). Thus, it is essential to assess the necessity of mass testing to have a number closer to reality for public health measures to be taken properly.

Undoubtedly, the SARS-CoV-2 RT-PCR testing is crucial since the knowledge of the etiological agent responsible for to cause of the SARI can direct the proper management of the disease, that is, to a suitable treatment that can prevent deaths and to adequate measures to decrease the SARS-CoV-2 spread.

### Limitations

We based our study on a public dataset, and the authors did not have access to the original data. Some patients' features had many patients without the data presentation, which reduced the study power. We cannot assure some patients with SARI with an undefined etiological agent are indeed patients with COVID-19 since we do not have a positive result in the SARS-CoV-2 RT-PCR test. In our study, we did not evaluate the information about COVID-19 vaccination, which could bias our results.

## Conclusions

In Brazil, the COVID-19 underreporting (here, namely, like patients with SARI due to an undefined etiological agent) might be associated with enhanced mortality, more evident in distinct social groups. The patients' features are unequal between patients' groups according to the SARI diagnoses and can be used to determine the risk of possible COVID-19 underreporting in our population. Patients with a higher risk of death had a different epidemiological profile than patients who recovered. Understanding the possible COVID-19 underreporting in Brazil is essential to take public health measures properly and attenuate the COVID-19 pandemic.

## Data Availability Statement

The datasets presented in this study can be found in online repositories. The names of the repository/repositories and accession number(s) can be found in the article/Supplementary Material.

## Ethics Statement

Ethical review and approval was not required for the study on human participants in accordance with the local legislation and institutional requirements. Written informed consent from the participants' legal guardian/next of kin was not required to participate in this study in accordance with the national legislation and the institutional requirements.

## Author Contributions

MB and FM made substantial contributions to the study conception and design, performed the acquisition, analysis, and interpretation of data for the work. NS, MB, and FM drafted the work and revised it critically for important intellectual content, and gave the final approval of the version to be published.

## Funding

MB Fundação de Amparo à Pesquisa do Estado de São Paulo (Foundation for Research Support of the State of São Paulo, Brazil; #2021/05810-7).

## Conflict of Interest

The authors declare that the research was conducted in the absence of any commercial or financial relationships that could be construed as a potential conflict of interest.

## Publisher's Note

All claims expressed in this article are solely those of the authors and do not necessarily represent those of their affiliated organizations, or those of the publisher, the editors and the reviewers. Any product that may be evaluated in this article, or claim that may be made by its manufacturer, is not guaranteed or endorsed by the publisher.
