# Supplementary material for: Epidemiologic Profile of Severe Acute Respiratory Infection in Brazil During the COVID-19 Pandemic: An Epidemiological Study
Source: Front Microbiol. 2022 Jul 1;13:911036. doi: 10.3389/fmicb.2022.911036 (PMC9288583; doi:10.3389/fmicb.2022.911036)
Supplement: Supplementary file 1 [file Data_Sheet_1.pdf]

Supplement

Title: Epidemiologic profile of severe acute respiratory infection in Brazil during the COVID-19 pandemic

Supplementary Table 1. Place of notification of the hospitalized patients due to severe acute respiratory infection (SARI) in Brazil during the Coronavirus Disease (COVID)-19 pandemic regarding the SARI categories.

State of notification	SARI due to Influenza virus	SARI due to other respiratory viruses' infection	SARI due to another known etiological agent	SARI due to an undefined etiological agent	SARI due to COVID-19 (SARS-CoV-2)	Total
Acre	12 (0.3%)	0 (0.0%)	2 (0.0%)	2,098 (0.2%)	4,528 (0.2%)	6,640 (0.2%)
Alagoas	15 (0.4%)	12 (0.1%)	22 (0.3%)	10,791 (1.2%)	20,928 (1.2%)	31,768 (1.2%)
Amazonas	23 (0.7%)	477 (2.9%)	145 (2.1%)	11,029 (1.2%)	38,957 (2.1%)	50,631 (1.8%)
Amapá	9 (0.3%)	12 (0.1%)	4 (0.1%)	586 (0.1%)	5,707 (0.3%)	6,318 (0.2%)
Bahia	245 (7.1%)	632 (3.8%)	439 (6.4%)	29,611 (3.3%)	69,810 (3.8%)	100,737 (3.7%)
Ceará	167 (4.8%)	287 (1.7%)	113 (1.6%)	47,319 (5.3%)	63,148 (3.5%)	111,034 (4.1%)
Federal District	47 (1.4%)	1,256 (7.6%)	58 (0.8%)	13,210 (1.5%)	43,019 (2.4%)	57,590 (2.1%)
Espírito Santo	38 (1.1%)	100 (0.6%)	159 (2.3%)	7,528 (0.8%)	14,564 (0.8%)	22,389 (0.8%)
Goiás	66 (1.9%)	486 (2.9%)	154 (2.2%)	19,413 (2.2%)	70,522 (3.9%)	90,641 (3.3%)
Maranhão	447 (12.9%)	31 (0.2%)	182 (2.7%)	12,201 (1.4%)	20,963 (1.2%)	33,824 (1.2%)
Minas Gerais	289 (8.3%)	605 (3.6%)	837 (12.2%)	119,181 (13.3%)	177,613 (9.8%)	298,525 (10.9%)
Mato Grosso do Sul	82 (2.4%)	586 (3.5%)	112 (1.6%)	15,037 (1.7%)	31,636 (1.7%)	47,453 (1.7%)
Mato Grosso	42 (1.2%)	30 (0.2%)	58 (0.8%)	8,581 (1.0%)	42,754 (2.4%)	51,465 (1.9%)
Pará	86 (2.5%)	102 (0.6%)	40 (0.6%)	21,290 (2.4%)	46,436 (2.6%)	67,954 (2.5%)
Paraíba	48 (1.6%)	7 (0.0%)	254 (3.7%)	15,299 (1.7%)	27,642 (1.5%)	43,250 (1.6%)
Pernambuco	180 (5.2%)	246 (1.5%)	42 (0.6%)	55,258 (6.2%)	49,358 (2.7%)	105,084 (3.8%)
Piauí	104 (3.0%)	197 (1.2%)	39 (0.6%)	7,681 (0.9%)	21,679 (1.2%)	29,700 (1.1%)
Paraná	105 (3.0%)	3,923 (23.6%)	144 (2.1%)	70,353 (7.9%)	118,535 (6.5%)	193,060 (7.0%)
Rio de Janeiro	187 (5.4%)	816 (4.9%)	281 (4.1%)	72,446 (8.1%)	173,093 (9.5%)	246,823 (9.0%)
Rio Grande do Norte	34 (1.0%)	51 (0.3%)	155 (2.3%)	7,645 (0.9%)	18,495 (1.0%)	26,380 (1.0%)
Rondônia	35 (1.0%)	13 (0.1%)	180 (2.6%)	5,189 (0.6%)	15,604 (0.9%)	21,021 (0.8%)
Roraima	9 (0.3%)	18 (0.1%)	10 (0.1%)	659 (0.1%)	4,143 (0.2%)	4,839 (5.4%)
Rio Grande do Sul	48 (1.4%)	1,236 (7.4%)	250 (3.6%)	36,153 (4.0%)	110,762 (6.1%)	148,449 (5.4%)
Santa Catarina	38 (1.1%)	695 (4.2%)	136 (2.0%)	22,212 (2.5%)	72,569 (4.0%)	95,650 (3.5%)
Sergipe	32 (0.9%)	57 (0.3%)	79 (1.2%)	8,208 (0.9%)	18,493 (1.0%)	26,869 (1.0%)
São Paulo	1,065 (30.7%)	4,649 (28.0%)	2,933 (42.7%)	267,536 (29.9%)	526,184 (29.0%)	802,367 (29.3%)
Tocantins	21 (0.6%)	103 (0.6%)	38 (0.6%)	9,693 (1.1%)	9,956 (0.5%)	19,811 (0.7%)

Individuals by place of notification per 1,000 inhabitants

State of notification	SARI due to Influenza virus	SARI due to other respiratory viruses' infection	SARI due to another known etiological agent	SARI due to an undefined etiological agent	SARI due to COVID-19 (SARS-CoV-2)	Total
Acre	0.01	<0.01	<0.01	2.38	5.13	7.53
Alagoas	<0.01	<0.01	0.01	3.23	6.27	9.52
Amazonas	0.01	0.12	0.03	2.66	9.40	12.22
Amapá	0.01	0.01	<0.01	0.69	6.75	7.47

Bahia	0.02	0.04	0.03	1.99	4.69	6.77
Ceará	0.02	0.03	0.01	5.18	6.91	12.16
Federal District	0.02	0.42	0.02	4.38	14.27	19.10
Espírito Santo	0.01	0.02	0.04	1.87	3.62	5.57
Goiás	0.01	0.07	0.02	2.77	10.05	12.91
Maranhão	0.06	<0.01	0.03	1.72	2.96	4.78
Minas Gerais	0.01	0.03	0.04	5.63	8.39	14.10
Mato Grosso do Sul	0.03	0.21	0.04	5.41	11.38	17.08
Mato Grosso	0.01	0.01	0.02	2.46	12.27	14.77
Pará	0.01	0.01	<0.01	2.47	5.40	7.90
Paraíba	0.01	<0.01	0.06	3.81	6.88	10.76
Pernambuco	0.02	0.03	<0.01	5.78	5.16	11.00
Piauí	0.03	0.06	0.01	2.35	6.62	9.07
Paraná	0.01	0.34	0.01	6.15	10.37	16.88
Rio de Janeiro	0.01	0.05	0.02	4.20	10.03	14.30
Rio Grande do Norte	0.01	0.01	0.04	2.18	5.27	7.52
Rondônia	0.02	0.01	0.10	2.92	8.78	11.83
Roraima	0.01	0.03	0.02	1.09	6.84	7.99
Rio Grande do Sul	<0.01	0.11	0.02	3.18	9.74	13.05
Santa Catarina	0.01	0.10	0.02	3.10	10.13	13.35
Sergipe	0.01	0.02	0.03	3.57	8.04	11.69
São Paulo	0.02	0.10	0.06	5.83	11.46	17.47
Tocantins	0.01	0.07	0.02	6.16	6.33	12.60

We presented the data as the number of individuals (N) and percentage (%).

Supplementary Table 2. Place of residence of the hospitalized patients due to severe acute respiratory infection (SARI) in Brazil during the Coronavirus Disease (COVID)-19 pandemic regarding the SARI classification

Place of residence	SARI due to Influenza virus	SARI due to other respiratory viruses' infection	SARI due to another known etiological agent	SARI due to an undefined etiological agent	SARI due to COVID-19 (SARS-CoV-2)	Total
Acre	12 (0.3%)	0 (0.0%)	2 (0.0%)	2,095 (0.2%)	4,529 (0.2%)	6,638 (0.2%)
Alagoas	16 (0.5%)	12 (0.1%)	22 (0.3%)	10,873 (1.2%)	21,055 (1.2%)	31,978 (1.2%)
Amazonas	24 (0.7%)	481 (2.9%)	148 (2.2%)	11,209 (1.3%)	39,861 (2.2%)	51,723 (1.9%)
Amapá	9 (0.3%)	12 (0.1%)	5 (0.1%)	602 (0.1%)	5,556 (0.3%)	6,184 (0.2%)
Bahia	244 (7.0%)	636 (3.8%)	439 (6.4%)	29,878 (3.3%)	70,075 (3.9%)	101,272 (3.7%)
Ceará	170 (4.9%)	288 (1.7%)	111 (1.6%)	47,199 (5.3%)	63,095 (3.5%)	110,863 (4.0%)
Federal District	45 (1.3%)	1,012 (6.1%)	47 (0.7%)	11,805 (1.3%)	39,597 (2.2%)	52,506 (1.9%)
Espírito Santo	40 (1.2%)	99 (0.6%)	158 (2.3%)	7,584 (0.8%)	14,648 (0.8%)	22,529 (0.8%)
Goiás	71 (2.0%)	723 (4.4%)	163 (2.4%)	20,761 (2.3%)	73,061 (4.0%)	94,779 (3.5%)
Maranhão	449 (13.0%)	45 (0.3%)	185 (2.7%)	12,579 (1.4%)	21,840 (1.2%)	35,098 (1.3%)
Minas Gerais	290 (8.4%)	627 (3.8%)	841 (12.3%)	119,273 (13.3%)	178,426 (9.8%)	299,457 (10.9%)
Mato Grosso do Sul	82 (2.4%)	587 (3.5%)	113 (1.6%)	15,128 (1.7%)	31,962 (1.8%)	47,872 (1.7%)
Mato Grosso	44 (1.3%)	36 (0.2%)	60 (0.9%)	8,692 (1.0%)	43,193 (2.4%)	52,025 (1.9%)
Pará	87 (2.5%)	104 (0.6%)	38 (0.6%)	21,547 (2.4%)	47,250 (2.6%)	69,026 (2.5%)
Paraíba	49 (1.4%)	8 (0.0%)	255 (3.7%)	15,336 (1.7%)	27,616 (1.5%)	43,264 (1.6%)
Pernambuco	177 (5.1%)	248 (1.5%)	43 (0.6%)	54,932 (6.1%)	49,346 (2.7%)	104,746 (3.8%)
Piauí	99 (2.9%)	183 (1.1%)	39 (0.6%)	7,352 (0.8%)	20,936 (1.2%)	28,609 (1.0%)
Paraná	101 (2.9%)	3,898 (23.5%)	142 (2.1%)	70,043 (7.8%)	118,600 (6.5%)	192,784 (7.0%)
Rio de Janeiro	188 (5.4%)	815 (4.9%)	286 (4.2%)	72,561 (8.1%)	173,251 (9.5%)	247,101 (9.0%)
Rio Grande do Norte	33 (1.0%)	52 (0.3%)	153 (2.2%)	7,697 (0.9%)	18,489 (1.0%)	26.424 (1.0%)
Rondônia	36 (1.0%)	14 (0.1%)	176 (2.6%)	5,184 (0.6%)	15,891 (0.9%)	21,301 (0.8%)
Roraima	9 (0.3%)	19 (0.1%)	9 (0.1%)	693 (0.1%)	4,197 (0.2%)	4,927 (0.2%)
Rio Grande do Sul	48 (1.4%)	1,239 (7.5%)	252 (3.7%)	36,235 (4.0%)	110,974 (6.1%)	148,748 (5.4%)
Santa Catarina	35 (1.0%)	704 (4.2%)	135 (2.0%)	22,338 (2.5%)	72,328 (4.0%)	95,540 (3.5%)
Sergipe	31(0.9%)	59 (0.4%)	78 (1.1%)	8,104 (0.9%)	18,283 (1.0%)	26,555 (1.0%)
São Paulo	1,057 (30.5%)	4,616 (27.8%)	2,925 (42.6%)	266,943 (29.8%)	522,841(28.8%)	798,382 (29.1%)
Tocantins	19 (0.5%)	101 (0.6%)	39 (0.6%)	9,479 (1.1%)	9,960 (0.5%)	19,598 (0.7%)

Individuals by place of notification per 1,000 inhabitants

State of notification	SARI due to Influenza virus	SARI due to other respiratory viruses' infection	SARI due to another known etiological agent	SARI due to an undefined etiological agent	SARI due to COVID-19 (SARS-CoV-2)	Total
Acre	0.01	<0.01	<0.01	2.38	5.14	7.53

Alagoas	<0.01	<0.01	0.01	3.26	6.31	9.58
Amazonas	0.01	0.12	0.04	2.70	9.62	12.48
Amapá	0.01	0.01	0.01	0.71	6.57	7.31
Bahia	0.02	0.04	0.03	2.01	4.71	6.81
Ceará	0.02	0.03	0.01	5.17	6.91	12.14
Federal District	0.01	0.34	0.02	3.92	13.13	17.41
Espírito Santo	0.01	0.02	0.04	1.89	3.65	5.61
Goiás	0.01	0.10	0.02	2.96	10.41	13.50
Maranhão	0.06	0.01	0.03	1.78	3.09	4.96
Minas Gerais	0.01	0.03	0.04	5.63	8.43	14.15
Mato Grosso do Sul	0.03	0.21	0.04	5.44	11.50	17.23
Mato Grosso	0.01	0.01	0.02	2.49	12.40	14.93
Pará	0.01	0.01	<0.01	2.50	5.49	8.02
Paraíba	0.01	<0.01	0.06	3.82	6.87	10.77
Pernambuco	0.02	0.03	<0.01	5.75	5.16	10.96
Piauí	0.03	0.06	0.01	2.25	6.40	8.74
Paraná	0.01	0.34	0.01	6.13	10.37	16.86
Rio de Janeiro	0.01	0.05	0.02	4.20	10.03	14.31
Rio Grande do Norte	0.01	0.01	0.04	2.19	5.27	7.53
Rondônia	0.02	0.01	0.10	2.92	8.94	11.99
Roraima	0.01	0.03	0.01	1.14	6.93	8.13
Rio Grande do Sul	<0.01	0.11	0.02	3.18	9.75	13.07
Santa Catarina	<0.01	0.10	0.02	3.12	10.09	13.33
Sergipe	0.01	0.03	0.03	3.53	7.95	11.55
São Paulo	0.02	0.10	0.06	5.81	11.39	17.39
Tocantins	0.01	0.06	0.02	6.03	6.33	12.46

We presented the data as the number of individuals (N) and percentage (%).

Supplementary Table 3. Odds ratio (OR) for the association between the severe acute respiratory infection (SARI) categories and the features of the hospitalized patients due to SARI in Brazil during the Coronavirus Disease (COVID)-19 pandemic.

Patient's features	Category	OR (95%CI) SARI due to an undefined etiological agent vs. COVID-19	OR (95%CI) SARI due to an undefined etiological agent vs. others [a]
Sex	Female	1.153 (1.147 to 1.159)	1.152 (1.146 to 1.158)
	Male	Reference	Reference
Age	<1 y.o.	15.100 (14.760 to 15.460)	8.028 (7.882 to 8.176)
	1-12 y.o.	17.660 (17.350 to 17.970)	12.090 (11.910 to 12.280)
	13-24 y.o.	3.201 (3.153 to 3.249)	3.122 (3.076 to 3.169)
	25-60 y.o.	Reference	Reference
	61-72 y.o.	1.179 (1.170 to 1.187)	1.178 (1.170 to 1.187)
	73-85 y.o.	1.587 (1.575 to 1.599)	1.583 (1.572 to 1.595)
	+85 y.o.	2.104 (2.083 to 2.125)	2.092 (2.072 to 2.113)
Race	White	Reference	Reference
	Black	1.311 (1.285 to 1.327)	1.313 (1.297 to 1.329)
	Asian	1.081 (1.053 to 1.110)	1.087 (1.059 to 1.116)
	Individuals from a multiracial background	1.214 (1.207 to 1.221)	1.214 (1.207 to 1.222)
	Indigenous peoples	1.210 (1.145 to 1.280)	1.198 (1.133 to 1.266)
Educational level	Illiterate	3.468 (3.400 to 3.537)	3.323 (3.258 to 3.389)
	1st fundamental cycle	2.035 (2.003 to 2.068)	2.023 (1.991 to 2.056)
	2nd fundamental cycle	1.571 (1.544 to 1.599)	1.564 (1.537 to 1.591)
	High school	1.244 (1.224 to 1.264)	1.244 (1.224 to 1.264)
	University education	Reference	Reference
	Not applicable	-	-
Place of residence	Urban	Reference	Reference
	Rural	1.228 (1.214 to 1.243)	1.227 (1.213 to 1.241)
	Peri-urban	1.420 (1.362 to 1.479)	1.307 (1.256 to1.361)
Living in a Flu outbreak region	Yes	0.772 (0.765 to 0.779)	0.779 (0.772 to 0.786)
	No	Reference	Reference
Received Flu vaccine	Yes	1.285 (1.263 to 1.297)	1.284 (1.273 to 1.296)
	No	Reference	Reference
Used antiviral drug to treat the clinical signs	Yes	1.462 (1.449 to 1.475)	1.440 (1.258 to 1.272)
	No	Reference	Reference
Intensive care unit	Yes	0.650 (0.646 to 0.654)	0.654 (0.650 to 0.658)
	No	Reference	Reference
Mechanical ventilatory support	Invasive	0.392 (0.388 to 0.395)	0.398 (0.394 to 0.401)
	Non-invasive	0.528 (0.524 to 0.531)	0.533 (0.529 to 0.536)
	Not required	Reference	Reference
Closure criterion	Laboratorial criterion	Reference	Reference
	Clinical - Epidemiological	1.831 (1.790 to 1.873)	1.835 (1.794 to 1.876)
	Clinical	3.520 (3.475 to 3.565)	3.504 (3.460 to 3.549)
	Clinical - Image exams	0.143 (0.139 to 0.147)	0.145 (0.141 to 0.149)
Outcome	Cure	Reference	Reference
	Death	0.487 (0.484 to 0.491)	0.495 (0.491 to 0.498)
	Death not related to SARI	14.790 (14.300 to 15.300)	12.580 (12.190 to 12.980)
Received SARS-CoV-2 vaccines	Yes	1.388 (1.372 to 1.403)	1.403 (1.388 to 1.419)
	No	Reference	Reference

[a], others included SARI due to Influenza, SARI due to another respiratory viruses' infection, SARI due to another known etiological agent, and SARI due to COVID-19 (SARS-CoV-2).
We presented the data as the number of individuals (N) and percentage (%).
We performed the statistical analyses using the chi-square test. We adopted an alpha error of 0.05.
95%CI, 95% confidence interval; SARS-CoV-2, Severe Acute Respiratory Syndrome Coronavirus 2; y.o., years old.

Supplementary Table 4. Multivariate analysis using the binary logistic regression compares patients with severe acute respiratory infection (SARI) due to an undefined etiological agent versus patients with Coronavirus Disease (COVID)-19.

Model 1. Included social and demographic data as well as hospitalization information [a]

**Patient's features**	**B**	**SE**	**Wald**	**df**	***P***	**OR**	**95%CI for OR**	
							**Lower**	**Upper**
Sex (female)	0.109	0.011	106.752	1	<0.001	1.116	1.093	1.139
Age								
<1 y.o.	2.022	0.072	779.967	1	<0.001	7.556	6.557	8.709
1-12 y.o.	2.214	0.041	2,907.583	1	<0.001	9.156	8.448	9.923
13-24 y.o.	1.273	0.028	2,036.079	1	<0.001	3.571	3.379	3.774
25-60 y.o.			5,743.692	6	<0.001			
61-72 y.o.	0.025	0.015	2.919	1	0.088	1.025	0.996	1.055
73-85 y.o.	0.363	0.016	493.049	1	<0.001	1.438	1.392	1.485
+85 y.o.	0.737	0.022	1,114.412	1	<0.001	2.091	2.002	2.183
Race								
White			261.985	4	<0.001			
Black	0.202	0.022	82.044	1	<0.001	1.224	1.172	1.279
Asian	0.007	0.053	0.020	1	0.888	1.008	0.908	1.118
Individuals from a multiracial background	-0.063	0.012	28.835	1	<0.001	0.939	0.918	0.961
Indigenous peoples	-1.099	0.096	130.515	1	<0.001	0.333	0.276	0.402
Educational level								
Illiterate	0.782	0.026	899.149	1	<0.001	2.186	2.077	2.301
1st fundamental cycle	0.620	0.019	1,012.349	1	<0.001	1.858	1.788	1.930
2nd fundamental cycle	0.473	0.020	543.038	1	<0.001	1.605	1.543	1.671
High school	0.220	0.019	135.261	1	<0.001	1.246	1.201	1.294
University education			1,539.346	5	<0.001			
Not applicable	0.766	0.059	166.155	1	<0.001	2.151	1.915	2.417
Place of residence								
Urban			101.672	2	<0.001			
Rural	0.228	0.023	100.430	1	<0.001	1.257	1.202	1.314
Peri-urban	0.118	0.092	1.646	1	0.200	1.126	0.939	1.349
Living in a Flu outbreak region	-0.348	0.013	707.729	1	<0.001	0.706	0.688	0.724
Received Flu vaccine	-0.071	0.011	39.241	1	<0.001	0.931	0.911	0.952
Used antiviral drugs to treat the disease	0.319	0.013	593.516	1	<0.001	1.376	1.341	1.412
Need for intensive care unit	-0.129	0.013	97.287	1	<0.001	0.879	0.857	.902
Closure criterion								
Laboratorial criterion			2,513.542	3	<0.001			
Clinical - Epidemiological	0.244	0.059	16.844	1	<0.001	1.277	1.136	1.434
Clinical	1.460	0.037	1,520.265	1	<0.001	4.307	4.002	4.635
Clinical - Image exams	-2.224	0.072	953.408	1	<0.001	0.108	0.094	0.125
Outcome								
Clinical cure			5,000.591	2	<0.001			
Death	-0.809	0.015	3,073.824	1	<0.001	0.445	0.433	0.458
Death not related to SARI	2.664	0.067	1,602.313	1	<0.001	14.350	12.595	16.349
Constant	-1.083	0.018	3,463.752	1	<0.001	0.339		

Model 2. Included social and demographic data [b]

Sex (female)	0.122	0.009	189.344	1	<0.001	1.130	1.110	1.150
Age								
<1 y.o.	1.887	0.063	890.149	1	<0.001	6.603	5.833	7.474
1-12 y.o.	2.052	0.035	3,433.696	1	<0.001	7.782	7.265	8.334
13-24 y.o.	1.051	0.023	2,107.818	1	<0.001	2.861	2.736	2.993
25-60 y.o.			6,185.885	6	<0.001			
61-72 y.o.	-0.114	0.012	87.579	1	<0.001	0.893	0.872	0.914
73-85 y.o.	0.099	0.014	53.973	1	<0.001	1.104	1.076	1.134
+85 y.o.	0.363	0.018	392.040	1	<0.001	1.438	1.387	1.490
Race								
White			327.497	4	<0.001			
Black	0.197	0.019	112.217	1	<0.001	1.218	1.175	1.264
Asian	-0.021	0.043	0.244	1	0.622	0.979	0.900	1.065

Individuals from a multiracial background	-0.046	0.010	23.103	1	<0.001	0.955	0.937	0.973
Indigenous peoples	-0.999	0.077	166.739	1	<0.001	0.368	0.317	0.429
Educational level								
Illiterate	0.702	0.021	1,068.117	1	<0.001	2.018	1.935	2.104
1st fundamental cycle	0.535	0.016	1,112.116	1	<0.001	1.707	1.654	1.761
2nd fundamental cycle	0.355	0.017	444.590	1	<0.001	1.426	1.380	1.474
High school	0.155	0.016	99.505	1	<0.001	1.167	1.132	1.204
University education			1,837.026	5	<0.001			
Not applicable	0.768	0.051	224.857	1	<0.001	2.156	1.950	2.384
Place of residence								
Urban			169.650	2	<0.001			
Rural	0.239	0.018	168.212	1	<0.001	1.270	1.225	1.317
Peri-urban	0.110	0.077	2.017	1	0.156	1.116	0.959	1.298
Living in a Flu outbreak region	-0.282	0.011	709.876	1	<0.001	0.755	0.739	0.770
Received Flu vaccine	0.021	0.010	4.827	1	0.028	1.021	1.002	1.040
Constant	-0.917	0.015	3,825.354	1	<0.001	0.400		

df, degrees of freedom; SE, standard error; OR, odds ratio; 95%CI, 95% confidence interval; y.o., years old. We adopted an alpha error of 0.05.

[a], we included the following patient's features in the statistical analysis: sex, age, race, educational level, place of residence, residence in a Flu outbreak region, Flu vaccine status during the last vaccination campaign, treatment for SARI symptoms with an antiviral drug, need for intensive care unit, closure criteria, and outcome.

[b], we included the following patient's features in the statistical analysis: sex, age, race, educational level, place of residence, residence in a Flu outbreak region, and Flu vaccine status during the last vaccination campaign.

Supplementary Table 5. Multivariate analysis using the binary logistic regression compares patients with severe acute respiratory infection (SARI) due to an undefined etiological agent versus patients with other causes of SARI [a].

**Patient's features**	**B**	**SE**	**Wald**	**df**	***P***	**OR**	**95%CI for OR**	
							**Lower**	**Upper**
**Model 1.** Included social and demographic data as well as hospitalization information [b]								
Sex (female)	0.106	0.010	102.727	1	<0.001	1.112	1.089	1.135
Age								
<1 y.o.	1.634	0.060	748.398	1	<0.001	5.123	4.557	5.759
1-12 y.o.	1.881	0.036	2,694.253	1	<0.001	6.563	6.113	7.046
13-24 y.o.	1.224	0.028	1,943.126	1	<0.001	3.401	3.221	3.591
25-60 y.o.			5,380.647	6	<0.001			
61-72 y.o.	0.026	0.015	3.277	1	0.070	1.027	0.998	1.056
73-85 y.o.	0.361	0.016	492.054	1	<0.001	1.435	1.390	1.481
+85 y.o.	0.728	0.022	1,097.236	1	<0.001	2.070	1.983	2.161
Race								
White			249.460	4	<0.001			
Black	0.202	0.022	83.437	1	<0.001	1.223	1.172	1.277
Asian	0.009	0.053	0.031	1	0.861	1.009	0.910	1.119
Individuals from a multiracial background	-0.064	0.012	30.481	1	<0.001	0.938	0.917	0.960
Indigenous peoples	-0.988	0.093	114.067	1	<0.001	0.372	0.310	0.446
Educational level								
Illiterate	0.771	0.026	890.418	1	<0.001	2.161	2.054	2.273
1st fundamental cycle	0.618	0.019	1,015.121	1	<0.001	1.855	1.786	1.927
2nd fundamental cycle	0.474	0.020	548.598	1	<0.001	1.606	1.544	1.671
High school	0.224	0.019	141.350	1	<0.001	1.252	1.206	1.299
University education			1,529.720	5	<0.001			
Not applicable	0.507	0.050	101.129	1	<0.001	1.660	1.504	1.833
Place of residence								
Urban			108.719	2	<0.001			
Rural	0.232	0.022	106.649	1	<0.001	1.261	1.207	1.318
Peri-urban	0.144	0.089	2.612	1	0.106	1.155	0.970	1.374
Living in a Flu outbreak region	-0.329	0.013	648.216	1	<0.001	0.720	0.702	0.738
Received Flu vaccine	-0.065	0.011	33.461	1	<0.001	0.937	0.917	0.958
Used antiviral drugs to treat the disease	0.272	0.013	443.500	1	<0.001	1.312	1.280	1.346
Need for intensive care unit	-0.133	0.013	106.311	1	<0.001	0.875	0.853	0.898
Closure criterion								
Laboratorial criterion			2,537.194	3	<0.001			
Clinical - Epidemiological	0.268	0.059	20.784	1	<0.001	1.307	1.165	1.467
Clinical	1.447	0.037	1,549.414	1	<0.001	4.252	3.956	4.570
Clinical - Image exams	-2.193	0.071	944.014	1	<0.001	0.112	0.097	0.128
Outcome								
Clinical cure			4,979.857	2	<0.001			
Death	-0.795	0.015	2,999.701	1	<0.001	0.452	0.439	0.465
Death not related to SARI	2.529	0.063	1,635.904	1	<0.001	12.546	11.099	14.182
Constant	-1.089	0.018	3,539.966	1	<0.001	0.336		
**Model 2.** Included social and demographic data [c]								
Sex (female)	0.119	0.009	183.068	1	<0.001	1.126	1.107	1.146
Age								
<1 y.o.	1.562	0.053	863.926	1	<0.001	4.766	4.295	5.289
1-12 y.o.	1.793	0.032	3,220.466	1	<0.001	6.007	5.646	6.390
13-24 y.o.	1.020	0.023	2,027.642	1	<0.001	2.773	2.652	2.899
25-60 y.o.			5,861.376	6	<0.001			
61-72 y.o.	-0.109	0.012	81.596	1	<0.001	0.896	0.875	0.918
73-85 y.o.	0.103	0.013	58.835	1	<0.001	1.109	1.080	1.139
+85 y.o.	0.363	0.018	395.729	1	<0.001	1.438	1.388	1.491
Race								
White			306.008	4	<0.001			
Black	0.196	0.018	112.900	1	<0.001	1.217	1.174	1.262
Asian	-0.020	0.043	0.218	1	0.640	0.980	0.901	1.066

Individuals from a multiracial background	-0.044	0.010	20.868	1	<0.001	0.957	0.940	0.975
Indigenous peoples	-0.908	0.075	147.422	1	<0.001	0.403	0.348	0.467
Educational level								
Illiterate	0.692	0.021	1,052.636	1	<0.001	1.997	1.915	2.082
1st fundamental cycle	0.532	0.016	1,105.440	1	<0.001	1.702	1.649	1.756
2nd fundamental cycle	0.354	0.017	445.223	1	<0.001	1.425	1.379	1.473
High school	0.157	0.015	103.124	1	<0.001	1.170	1.135	1.206
University education			1,792.379	5	<0.001			
Not applicable	0.512	0.044	133.933	1	<0.001	1.669	1.530	1.820
Place of residence								
Urban			179.807	2	<0.001			
Rural	0.243	0.018	177.879	1	<0.001	1.276	1.231	1.322
Peri-urban	0.122	0.075	2.621	1	0.105	1.130	0.975	1.309
Living in a Flu outbreak region	-0.267	0.010	651.564	1	<0.001	0.766	0.750	0.781
Received Flu vaccine	0.022	0.009	5.626	1	0.018	1.023	1.004	1.042
Constant	-0.931	0.015	3,969.279	1	<0.001	0.394		

df, degrees of freedom; SE, standard error; Sig, statistical significance (P-value); OR, odds ratio; 95%CI, 95% confidence interval; y.o., years old. We adopted an alpha error of 0.05.

[a], others included SARI due to Influenza, SARI due to another respiratory infection, SARI due to another known etiological agent, SARI due to COVID-19 (SARS-CoV-2).

[b], we included the following patient's features in the statistical analysis: sex, age, race, educational level, place of residence, residence in a Flu outbreak region, Flu vaccine status during the last vaccination campaign, treatment for SARI symptoms with an antiviral drug, need for intensive care unit, closure criteria, and outcome.

[c], we included the following patient's features in the statistical analysis: sex, age, race, educational level, place of residence, residence in a Flu outbreak region, and Flu vaccine status during the last vaccination campaign.
